# Explainable Multimodal Machine Learning Predicts 90-Day Treatment Failure in Older Patients with Fragility Fractures of the Pelvis

**DOI:** 10.3390/jcm15166487

**Published:** 2026-08-21

**Authors:** Kangwei Wang, Yulin Cao, Nan Gao, Cong Ma, Jianwen Wang, Zishen Xia, Aiwen Gui, Yong Liu, Yuxiong Weng

**Affiliations:** 1 Department of Hand Surgery, Union Hospital, Tongji Medical College, Huazhong University of Science and Technology, Wuhan 430022, China; m202476420@hust.edu.cn (K.W.); d202382051@hust.edu.cn (N.G.); tjmu71union@hust.edu.cn (C.M.); d202582489@hust.edu.cn (J.W.); m202376255@hust.edu.cn (Z.X.); m202576459@hust.edu.cn (A.G.); 2 Department of Orthopedic Surgery, Wuhan Fourth Hospital, Puai Hospital, Tongji Medical College, Huazhong University of Science and Technology, Wuhan 430033, China; caoyulin1220@126.com; 3 Department of Orthopedics, Union Hospital, Tongji Medical College, Huazhong University of Science and Technology, Wuhan 430022, China

**Keywords:** fragility fractures of the pelvis (FFP), 90-day treatment failure (TF90), machine learning, fracture morphology, bone metabolism

## Abstract

**Background**: Fragility fractures of the pelvis (FFP) are increasingly encountered in older adults, yet early deterioration is difficult to anticipate because fracture instability interacts with frailty and systemic vulnerability. We developed and validated an admission-based multimodal framework to predict 90-day treatment failure (TF90) before definitive management. **Methods**: This multicentre retrospective prediction study included 1684 consecutive patients aged ≥65 years with FFP treated at five tertiary hospitals. TF90 was defined as persistent fracture-related pain or immobility, delayed conversion to operative stabilisation, secondary displacement, FFP-related unplanned readmission, revision or unplanned reoperation, or all-cause mortality within 90 days. Only predictors available within 24 h of admission and before the definitive treatment decision were eligible, including CT-defined fracture morphology, frailty, clinical characteristics and routine laboratory biomarkers; DXA and specialised bone metabolism measurements were evaluated separately in an extended model. Four prespecified models were developed in 985 patients, temporally validated in 520 patients and evaluated in a completely held-out Centre E internal–external validation cohort of 179 patients, with additional leave-one-centre-out internal–external cross-validation. **Results**: TF90 occurred in 307 patients (18.2%) and increased from 8.1% in FFP I to 37.1% in FFP IV. Higher risk was associated with advanced age, greater frailty, impaired prefracture mobility, bilateral posterior ring injury, greater displacement, systemic inflammation, hypoalbuminaemia and renal dysfunction. In temporal validation, AUROCs were 0.732 for the simple logistic model, 0.763 for the core logistic model, 0.759 for the core random forest and 0.755 for the extended random forest. Neither greater algorithmic complexity nor specialised skeletal measurements provided reproducible incremental value. A development-derived high-risk stratum had a TF90 incidence of 32.2% and contained 70.0% of all events. At the fixed threshold of 0.209, sensitivity was 71.3%, specificity 70.0% and negative predictive value 93.1%. **Conclusions**: Pretreatment integration of pelvic ring mechanics, frailty and routinely available systemic biomarkers enables clinically relevant enrichment of older patients at risk of TF90. The model is best positioned to support intensified surveillance and structured reassessment rather than determine operative treatment. Independent prospective external validation, recalibration and clinical impact evaluation are required before routine implementation.

## 1. Introduction

Fragility fractures of the pelvis (FFP) have become a defining injury of population ageing and an increasing challenge in geriatric orthopaedic trauma [1,2,3]. Unlike high-energy pelvic ring disruptions, these fractures usually follow a low-energy fall [4,5,6]. Initial radiographs can appear deceptively reassuring, whereas computed tomography (CT) frequently reveals posterior ring disruption, bilateral injury or occult instability [7]. Clinical trajectories are heterogeneous [8]. Some patients recover with analgesia, mobilisation and rehabilitation; others remain unable to stand, develop progressive displacement, return unexpectedly to hospital or require delayed operative stabilisation [6,9,10]. The central challenge is not simply to identify the fracture, but to recognise at admission which apparently manageable injuries are likely to follow an unfavourable course [9].

Fracture morphology remains fundamental to FFP assessment [3,6,11]. The Rommens–Hofmann classification and systematic CT evaluation of the posterior pelvic ring provide an anatomical framework for characterising instability and informing management [6,12]. Nevertheless, anatomy captures only one dimension of risk [13,14]. Patients with similar fracture configurations may experience different outcomes according to physiological reserve, comorbidity, nutritional status, inflammatory activity, skeletal health and prefracture function [15,16]. Frailty is particularly relevant because it reflects vulnerability to acute stress, deconditioning, impaired rehabilitation tolerance, readmission and mortality beyond that conveyed by age or bone density alone [17,18]. The Clinical Frailty Scale (CFS) offers a structured measure of prefracture vulnerability, whereas inflammatory and nutritional indices characterise complementary aspects of systemic reserve [19,20,21]. Bone fragility and physiological vulnerability often coexist, but they are not interchangeable [22,23]. FFP should therefore be understood as a geriatric fracture syndrome in which mechanical instability interacts with host susceptibility [16].

No single conventional endpoint adequately captures early failure of an initial FFP management strategy [10]. Mortality reflects only the most severe consequence, whereas radiographical progression, delayed surgery, unplanned readmission and persistent loss of mobility represent distinct pathways of deterioration [9]. We therefore defined 90-day treatment failure (TF90) as at least one of six prespecified events: persistent fracture-related pain or inability to mobilise, delayed conversion from non-operative management to operative stabilisation, secondary displacement or morphological progression, FFP-related unplanned readmission, revision surgery or unplanned reoperation, or all-cause mortality [9]. Because these components differ in mechanism, objectivity and severity, and may overlap within the same patient, the composite requires component-wise reporting and sensitivity analyses using progressively more objective and severe endpoints [24]. This preserves clinical breadth without implying that pain, reoperation and death are equivalent events.

A clinically useful prognostic model must answer a precisely timed question. For admission-based prediction, only information available before the definitive management decision can legitimately inform risk estimation [25]. Incorporating treatment strategy, mobilisation response, length of stay, discharge destination or subsequent events would introduce temporal or outcome leakage and produce performance unavailable at the intended point of use [26]. A defensible framework therefore requires an explicit prediction landmark, strict separation of baseline predictors from subsequent care, and fold-specific handling of missing data [27]. Evaluation should extend beyond discrimination to include precision–recall performance, calibration of absolute risk, probabilistic accuracy and decision curve analysis (DCA) [28,29]. Temporal validation and internal–external cross-validation (IECV) should assess robustness and transportability within the study system, not substitute for independent external validation [30].

Information available at admission spans complementary domains. Posterior ring disruption, bilateral injury, displacement and fracture configuration reflect mechanical instability; frailty, comorbidity and prefracture function represent physiological reserve; and inflammatory, nutritional, haematological and renal indices characterise systemic vulnerability [31,32]. Dual-energy X-ray absorptiometry (DXA) and specialised bone-metabolism tests may add information, but they are not consistently available during acute admission and may have substantial missingness [33]. Their incremental value beyond routine clinical, imaging and laboratory information is uncertain. Increased algorithmic complexity should likewise not be assumed to improve prediction [34,35]. Machine-learning models must be compared directly with appropriately specified penalised regression and judged across discrimination, calibration, precision–recall performance and probabilistic accuracy [36]. The relevant question is whether complexity provides reproducible, clinically meaningful information beyond a transparent benchmark [36].

The conceptual innovation of this study lies in aligning a clinically coherent failure endpoint, an explicit pretreatment prediction landmark and a multidomain risk architecture within a leakage-resistant validation framework. We developed an admission-based multimodal approach to predict TF90 after FFP, positioning the landmark after baseline CT and the initial clinical and laboratory assessment, within 24 h of admission and before the definitive treatment decision. We compared a parsimonious logistic model, a comprehensive core logistic model, a core random forest (RF) and an extended RF incorporating DXA and specialised bone metabolism measurements. The primary composite, its individual components and prespecified objective, major clinical event and hard procedural-or-death endpoints were evaluated using grouped internal resampling, temporal validation, held-out-centre assessment and leave-one-centre-out IECV.

We hypothesised that integrating pelvic ring mechanics—including a study-specific Pelvic Ring Instability (PRI) score—with frailty and routinely available indicators of systemic vulnerability would improve prognostic stratification beyond a parsimonious admission benchmark. We also examined whether specialised skeletal measurements and greater algorithmic complexity provided reproducible incremental value. Rather than positioning the model as an automated determinant of operative treatment, we evaluated its potential to identify patients who may warrant closer surveillance, structured reassessment or earlier multidisciplinary review. Independent prospective external validation, recalibration and formal clinical impact evaluation remain necessary before routine use.

## 2. Materials and Methods

### 2.1. Study Design, Setting and Ethics

This multicentre retrospective prediction study included consecutive patients with FFP treated at five tertiary hospitals in Wuhan, China. For consistent reporting and analysis, the participating institutions were designated as follows: Centre A, Union Hospital, Tongji Medical College, Huazhong University of Science and Technology; Centre B, Tongji Hospital, Tongji Medical College, Huazhong University of Science and Technology; Centre C, Liyuan Hospital, Huazhong University of Science and Technology; Centre D, Wuhan Central Hospital; and Centre E, Wuhan Jinyintan Hospital. These centre designations were used thereafter throughout the analytical workflow, figures and tables. Eligible admissions occurred between 1 January 2015 and 31 December 2025. The clinical data used in this study were generated during routine patient care. Accordingly, clinical records predating the initial ethics approval had already been created as part of standard clinical practice. Following initial ethics approval, the investigators were authorised, in accordance with the approved protocol, to retrospectively access and analyse eligible routinely collected clinical records. No research-specific intervention or prospective experimental data collection was undertaken before ethics approval. Follow-up for the final potentially eligible admission was completed on 31 March 2026, the database was locked on 15 April 2026, and formal analyses commenced on 1 May 2026. The index admission was defined as the first hospitalisation during which FFP was confirmed, and the initial management strategy was established. The study was reported in accordance with STROBE principles [37]. The study protocol was initially approved by the Medical Ethics Committee of Tongji Medical College, Huazhong University of Science and Technology (approval No. TJ-IRB20230108) on 18 January 2023. The protocol subsequently underwent periodic continuing review by the Institutional Ethics Committee, with the most recent continuing review document dated 20 May 2026. Given the retrospective design and the use of de-identified routinely collected clinical data, the requirement for written informed consent was waived by the ethics committee. All patient data were de-identified before analysis and handled in accordance with institutional requirements for patient privacy and data protection. The study was conducted in accordance with the principles of the Declaration of Helsinki and the conditions specified in the ethics approval.

### 2.2. Data Sources, Case Identification and Eligibility

Potentially eligible admissions were identified from electronic health records, radiology archives, laboratory information systems, operative records, discharge-diagnosis databases, outpatient documentation and structured follow-up records. Case identification combined pelvic fracture diagnostic codes, low-energy trauma descriptors, FFP-related imaging terminology, orthopaedic admission records and relevant procedural codes. Duplicate records, repeated admissions and interhospital transfers were reconciled before eligibility assessment. Patients were eligible if they were aged 65 years or older; had a low-energy or osteoporotic pelvic fracture compatible with FFP; were admitted to an orthopaedic service at a participating centre; underwent an interpretable baseline pelvic CT examination before the prediction landmark; and had sufficient information to establish eligibility and ascertain treatment and vital status through 90 days. Exclusion criteria were high-energy or non-fragility pelvic injury; pathological fracture caused by malignancy or infection; previous pelvic surgery or pelvic malignancy; periprosthetic fracture; polytrauma requiring a separate major trauma pathway; duplicate or non-index admission; transfer before completion of baseline assessment; absence of an interpretable pretreatment CT examination; chronology incompatible with pretreatment prediction; unascertainable 90-day status; or insufficient information to determine TF90 [32]. Missing DXA or specialised bone metabolism measurements alone did not result in exclusion [38,39].

### 2.3. Prediction Landmark and Prevention of Information Leakage

The prediction landmark was defined separately for each patient as the time immediately after completion of baseline CT and the initial clinical and laboratory assessment, no later than 24 h after admission and before the first definitive treatment decision [25]. Temporal ordering was established using CT acquisition or reporting times, laboratory result availability, the first documented management plan, operative consent, procedure requests and definitive treatment orders. Only information available by the prediction landmark was eligible for modelling. Initial treatment strategy, treatment indication, osteoporosis treatment initiated during the index admission, mobilisation response, length of stay, discharge destination, subsequent procedures, readmissions and all outcome information were excluded from model inputs. Initial treatment was retained only for descriptive and prespecified stratified analyses. Cohort assignment preceded imputation, encoding, model fitting, hyperparameter tuning, probability recalibration, threshold determination and risk-stratum definition. Neither predictor distributions nor outcomes from the validation cohorts were used to modify the development pipeline.

### 2.4. Outcome Definitions and Ascertainment

The primary outcome was TF90, defined as the occurrence of at least one of six events within 90 days of the index admission: persistent fracture-related pain or immobility; delayed conversion from non-operative management to operative stabilisation; secondary displacement or progression to a more unstable FFP pattern; FFP-related unplanned readmission; revision surgery or unplanned reoperation; or all-cause mortality [9]. Persistent pain or immobility was assessed from day 14 to day 90 and was defined as fracture-attributable pain with an NRS score of at least 5 despite optimised analgesia, or inability attributable to FFP to stand, transfer or walk 10 m using the usual mobility aid for at least 48 h [10,40]. Delayed conversion was defined as unplanned operative stabilisation after initial non-operative management because of persistent symptoms, failure to mobilise or radiographical progression [9]. Secondary displacement was defined as new or increased pelvic ring displacement of at least 2 mm relative to baseline CT, or progression to a higher FFP category [41]. FFP-related readmission required an unplanned hospitalisation lasting more than 24 h and primarily attributable to fracture-related pain, mobility failure, radiographical progression, rehabilitation failure, or a wound- or implant-related complication [42]. Revision or reoperation comprised any unplanned repeat pelvic procedure after the index operation or delayed conversion [43]. Mortality comprised death from any cause within 90 days [44]. Outcome information was obtained from inpatient records, operative reports, radiology reports, discharge summaries, outpatient records, readmission documentation, structured telephone follow-up and mortality records. Potential events were independently reviewed by two trained clinical assessors blinded to model predictions, with disagreements resolved by consensus or senior orthopaedic adjudication. TF90 was analysed as an unweighted binary composite. Components were non-mutually exclusive and were also reported individually. For time-to-first-event analyses, the earliest qualifying event determined the event date; same-day events were resolved according to a prespecified severity hierarchy. Three sensitivity endpoints were evaluated: an objective composite excluding persistent pain or immobility; a major clinical event composite comprising delayed conversion, readmission, revision or reoperation, and mortality; and a hard procedural-or-death endpoint comprising delayed conversion, revision or reoperation, and mortality. A radiographical sensitivity analysis applied a stricter displacement threshold of at least 5 mm or progression to a higher FFP category.

### 2.5. Imaging Assessment, PRI and Frailty

Baseline pelvic radiographs and CT examinations were reviewed in axial, coronal and sagittal planes, with three-dimensional reconstructions considered when available. Recorded morphological characteristics included FFP category and subtype, posterior ring involvement, bilateral posterior ring involvement, pubic-ramus and sacral-ala fracture patterns, Denis sacral zone, H- or U-shaped sacral morphology, acetabular involvement, L5 transverse process fracture and maximum pelvic ring displacement. Maximum displacement was measured at the most displaced anterior or posterior ring component on the reconstruction demonstrating the greatest separation. Key morphological variables were independently assessed by two trained imaging reviewers blinded to TF90 status and model predictions, with disagreements resolved by consensus or senior musculoskeletal trauma adjudication. PRI was calculated exclusively from pretreatment CT as a study-specific ordinal summary of pelvic ring mechanical instability and ranged from 0 to 10. The score integrated five morphological features: FFP category (I–IV, 0–3 points), posterior ring involvement (0 or 2 points), bilateral posterior ring involvement (0 or 2 points), maximum displacement (<2.0 mm, 2.0–4.9 mm or ≥5.0 mm; 0, 1 or 2 points, respectively), and H- or U-shaped sacral morphology (0 or 1 point). Higher scores represented greater overall mechanical instability. The component weights provided an ordinal representation of increasing fracture complexity and instability rather than estimates of independent effect size. Because PRI summarises morphological features that are also represented individually in the imaging dataset, it was interpreted as an integrated morphology measure rather than as an independent anatomical characteristic. To examine the robustness of model performance to alternative representations of pelvic ring instability, a sensitivity analysis compared two otherwise identical RF specifications. In one specification, mechanical instability was represented by the composite PRI score; in the other, PRI was replaced by its five constituent variables—FFP type, posterior ring involvement, bilateral posterior ring involvement, maximum fracture displacement and H- or U-shaped sacral morphology. All other predictors, preprocessing procedures and RF hyperparameters were kept unchanged, allowing the effect of the alternative morphology representation to be evaluated directly. CFS was used to characterise the patient’s usual functional state approximately two weeks before fracture rather than their acute appearance at admission. Assessment integrated patient interview, collateral information from relatives or caregivers and pre-admission clinical and functional records. Assessors received structured training using standardised guidance, worked examples and calibration cases before data abstraction. A centre- and year-stratified subset of patient records was independently assessed by two trained raters, and inter-rater agreement was quantified using quadratic-weighted Cohen’s κ, an absolute agreement intraclass correlation coefficient, exact agreement and agreement within one CFS point.

### 2.6. Clinical, Laboratory and Skeletal Health Predictors

Clinical predictors available by the landmark included age, sex, body mass index, prefracture living status, preinjury mobility, CCI, ASA physical status class, CFS, injury mechanism, time from injury to admission, admission pain score, known osteoporosis, preinjury anti-osteoporosis treatment, and anticoagulant or antiplatelet use. For repeated laboratory measurements, the earliest valid result available by the prediction landmark was retained. Haematological and inflammatory variables included haemoglobin; white blood cell, neutrophil, lymphocyte and platelet counts; NLR; PLR; CRP; ESR; albumin; and D-dimer. Renal and metabolic variables included creatinine, eGFR, glucose, sodium, calcium, phosphate and alkaline phosphatase. The extended predictor set additionally included DXA T-score, 25(OH)D, parathyroid hormone, osteocalcin, PINP and β-CTX. Post-treatment measurements were ineligible. Units were harmonised before analysis, and implausible values or potential unit inconsistencies were verified against source records. Predictor inclusion was based on availability at the landmark and clinical relevance rather than univariable statistical significance.

### 2.7. Cohort Partitioning and Validation Framework

Patients admitted to Centres A–D between 2015 and 2022 formed the development cohort. Patients admitted to the same four centres between 2023 and 2025 formed the temporal validation cohort. Centre E was withheld in its entirety across 2015–2025 and served as the fixed held-out-centre internal–external validation cohort. Centre E contributed no information to preprocessing, model selection, tuning, recalibration, threshold selection or risk-stratum definition. Leave-one-centre-out IECV was additionally performed across Centres A–E. During each iteration, one complete centre was withheld, the modelling pipeline was developed using the remaining centres, and performance was evaluated in the omitted centre. Temporal validation, fixed Centre E validation and leave-one-centre-out IECV assessed complementary aspects of temporal stability and within-network transportability. These analyses were not considered independent external validation.

### 2.8. Missing Data and Preprocessing

Predictor missingness was quantified overall and according to centre, treatment year, cohort assignment and TF90 status. Patients remained eligible when individual predictors were missing, provided that eligibility and TF90 status could be established. All preprocessing was nested within model development. Within each training fold, continuous variables were imputed using the fold-specific median and categorical variables using the fold-specific most frequent category. Missingness indicators were retained for variables with clinically relevant patterns of unavailability. Categorical encoding, unit harmonisation and scaling required for penalised regression were fitted exclusively within each training fold and applied unchanged to the corresponding assessment fold. Validation observations and outcomes did not contribute to imputation, encoding, scaling, feature transformation, hyperparameter selection, recalibration, threshold determination or risk-group definition.

### 2.9. Model Development, Sample Size and Hyperparameter Tuning

Four models were prespecified: a parsimonious L2-penalised logistic-regression benchmark based on a restricted set of routinely available admission variables; a core L2-penalised logistic-regression model incorporating the complete core predictor set; a core RF using the same clinical, imaging and routine laboratory information; and an extended RF additionally incorporating DXA and bone metabolism measurements. The core logistic model and core RF were treated as complementary primary candidates. The core RF was selected for threshold-based stratification, DCA and model interpretation analyses because it accommodated non-linearities and interactions without requiring their explicit specification. Class weighting was used to address outcome imbalance. Sample size was determined by all eligible admissions during the prespecified study period; no a priori sample size calculation governed cohort inclusion. Model complexity was constrained using L2 penalisation, restricted tree depth, minimum terminal node size and grouped resampling. Hyperparameters were selected exclusively within the development cohort using fivefold grouped cross-validation. Centre–treatment year combinations were retained as intact groups so that patients treated at the same centre during the same calendar year were not divided between training and assessment folds. Mean cross-validated AUPRC was the primary tuning criterion, with mean AUROC used as the tie-breaker. The selected regularisation parameters were C = 0.05 for the parsimonious logistic model and C = 0.005 for the core logistic model. The core RF comprised 300 trees, a maximum depth of 4, a minimum terminal node size of 8, square-root feature sampling and balanced subsampling. The extended RF comprised 300 trees, a maximum depth of 6, a minimum terminal node size of 8 and square-root feature sampling. Development out-of-fold predictions were generated such that each patient’s probability was obtained from a model that had not been trained using that patient.

### 2.10. Recalibration, Operating Threshold and Risk Strata

Platt recalibration was fitted exclusively to development out-of-fold predictions and their corresponding outcomes. The resulting calibration function was applied unchanged to the temporal validation and Centre E cohorts. The core RF operating threshold of 0.209 was derived exclusively from development out-of-fold predictions using the prespecified threshold selection procedure recorded in the analysis code. It was fixed before validation and was not re-optimised using either validation cohort. Threshold performance was summarised using sensitivity, specificity, PPV, NPV, F1 score, the proportion of patients classified as positive and the proportion of TF90 events captured. Low-, intermediate- and high-risk groups were defined using tertiles of the development out-of-fold predictions, corresponding to cut points of 0.116 and 0.221. These boundaries were applied unchanged to the validation cohorts. The threshold and risk groups were intended for prognostic enrichment and surveillance assessment rather than as indications for operative treatment.

### 2.11. Model-Performance, Decision and Interpretability Analyses

Model discrimination was assessed using the AUROC and the AUPRC, with AUPRC interpreted in relation to the event prevalence of the corresponding cohort. Overall probabilistic accuracy was quantified using the Brier score. Calibration was evaluated using the calibration intercept, calibration slope, observed-to-expected ratio and grouped calibration plots, and was additionally summarised by centre, treatment year and initial treatment stratum. Ninety-five per cent confidence intervals were estimated using non-parametric patient-level bootstrap resampling. Differences in AUROC, AUPRC and Brier score between models were quantified using paired bootstrap resampling of the same patients. The incremental predictive value of the core models was evaluated relative to the parsimonious logistic-regression benchmark, whereas the contribution of specialised skeletal-health measurements was assessed by comparing the extended RF with the core RF. Robustness to alternative representations of pelvic ring instability was examined in a dedicated sensitivity analysis comparing two otherwise identical RF specifications. In the PRI-score specification, mechanical instability was represented by the composite PRI score, with its five constituent variables excluded. In the component-based specification, PRI was excluded and FFP type, posterior ring involvement, bilateral posterior ring involvement, maximum fracture displacement and H- or U-shaped sacral morphology were entered separately. The two representations were therefore mutually exclusive (Supplementary Figure S1). All other predictors, preprocessing procedures, cohort assignments and RF hyperparameters were held constant. Performance was evaluated in the temporal validation and Centre E internal–external validation cohorts using AUROC, AUPRC, Brier score and calibration measures, with paired bootstrap resampling used to quantify differences between the two specifications. DCA was used to estimate net benefit across threshold probabilities of 0.05–0.40, with each model compared against treat-all and treat-none strategies. The decision context represented prioritisation for closer surveillance, earlier reassessment, repeat imaging or multidisciplinary review rather than automatic treatment escalation. Permutation importance was quantified as the mean reduction in validation AUROC after random permutation of each predictor. Because permutation-based importance can be affected by correlation among predictors, these estimates were interpreted as contributions to the fitted prediction function rather than as independent effect estimates. Predicted-risk gradients and Spearman correlations were additionally used to characterise relationships between predicted risk and representative mechanical, frailty, inflammatory, nutritional and skeletal variables. These analyses were descriptive and were not interpreted causally. Prespecified subgroup analyses examined model performance according to sex, age, FFP category and initial treatment strategy. Component-specific analyses and analyses of alternative sensitivity endpoints were exploratory. A separate mortality model was not fitted because of the limited number of mortality events.

### 2.12. Statistical Analysis

Continuous variables were summarised as mean and standard deviation when approximately symmetrically distributed and as median and interquartile range when skewed or ordinal. Categorical variables were reported as number and percentage. Absolute SMDs were used to quantify between-group imbalance, with values of at least 0.10 considered potentially meaningful. Exploratory comparisons between patients with and without TF90 used the two-sided Mann–Whitney U test for continuous or ordinal variables and Pearson’s χ^2^ test or Fisher’s exact test for categorical variables, as appropriate. Spearman rank correlation was used to assess ordinal, skewed or monotonic associations. Baseline comparisons were descriptive and were not used to select model predictors. All conventional tests were two-sided, with α = 0.05. Exact *p*-values were reported to three decimal places, and values below 0.001 were reported as *p* < 0.001. No multiplicity adjustment was applied to exploratory baseline, subgroup, component-specific or model interpretation analyses. Analyses were performed using Python 3.13 and scikit-learn 1.8.0, with the random seed fixed at 20260628. The reproducibility archive contained the data dictionary, cohort assignments, complete predictor specifications, preprocessing pipelines, grouped cross-validation assignments, hyperparameter grids, development out-of-fold predictions, calibration functions, threshold selection outputs, bootstrap indices and validation predictions. All data cleaning, cohort partitioning, preprocessing, model development and validation procedures were completed using version-controlled analytical scripts. Model specifications and analytical decisions were fixed before evaluation of the temporal validation and Centre E cohorts. Results from subgroup, component-specific, interpretability and decision curve analyses were regarded as exploratory unless otherwise specified.

## 3. Results

### 3.1. Cohort Assembly, Temporal Patterns and TF90 Burden

**Figure 1:**
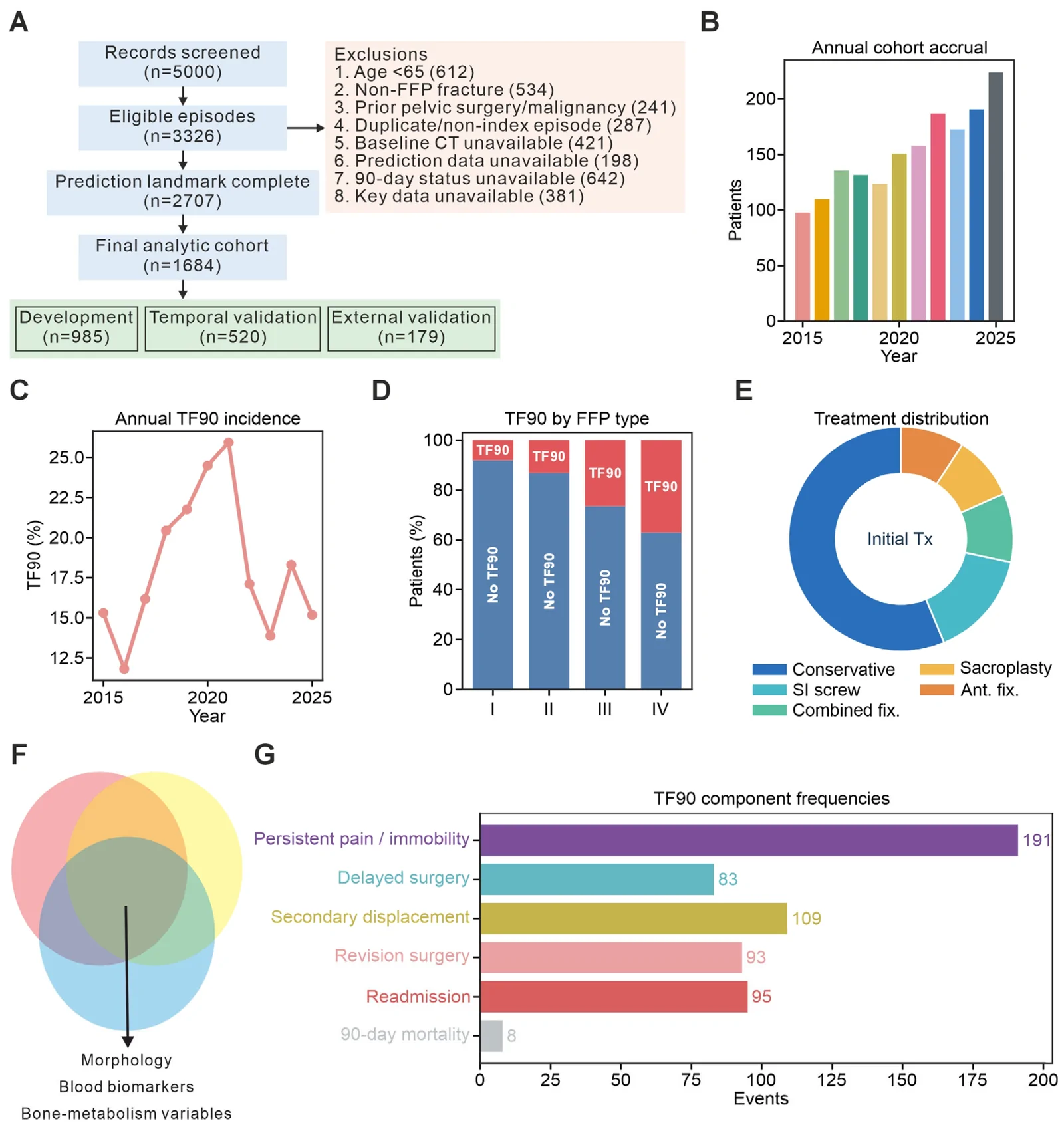
Cohort assembly, study architecture and burden of TF90. (**A**) Study flow from 5000 screened records to the final analytical cohort of 1684 patients, followed by allocation to the development cohort (n = 985), temporal validation cohort (n = 520) and completely held-out Centre E internal–external validation cohort (n = 179). (**B**) Annual patient accrual from 2015 to 2025. (**C**) Observed annual incidence of TF90. (**D**) Proportions of patients with and without TF90 across FFP categories I–IV. (**E**) Distribution of initial management strategies, comprising conservative management, percutaneous sacroiliac screw fixation, sacroplasty, anterior ring fixation, combined anterior–posterior fixation and lumbopelvic fixation. (**F**) Admission predictor domains represented in the modelling framework, including fracture morphology, routinely available blood biomarkers and specialised bone metabolism measurements included in the extended model. (**G**) Frequencies of the six prespecified TF90 components. FFP, fragility fractures of the pelvis; TF90, 90-day treatment failure.

Of 5000 records screened across the five participating centres, 3326 met the episode-level eligibility criteria, 2707 were evaluable at the admission prediction landmark and 1684 patients comprised the final analytical cohort, including 985 in development, 520 in temporal validation and 179 in the fully held-out Centre E internal–external validation cohort (Figure 1A). The complete sequential screening process, including all exclusion categories, is reported in Supplementary Table S1; the principal exclusions were unascertainable 90-day treatment or vital status (n = 642), age <65 years (n = 612), high-energy, pathological or non-FFP fractures (n = 534), absence of an interpretable baseline CT before the prediction landmark (n = 421), and insufficient eligibility- or outcome-defining information (n = 381). Annual accrual increased from 98 patients in 2015 to 224 in 2025 (Figure 1B), whereas annual TF90 incidence ranged from 11.8% to 25.9%, peaking in 2021 before declining to 15.2% in 2025 (Figure 1C). TF90 incidence increased stepwise with fracture severity, from 8.1% in FFP I and 13.1% in FFP II to 26.5% in FFP III and 37.1% in FFP IV (Figure 1D). Initial management was conservative in 908 patients (53.9%); operative strategies comprised percutaneous sacroiliac screw fixation in 249 (14.8%), combined anterior–posterior fixation in 160 (9.5%), sacroplasty in 149 (8.8%), anterior ring fixation in 148 (8.8%) and lumbopelvic fixation in 70 (4.2%) (Figure 1E). The admission prediction framework integrated clinical and frailty characteristics, fracture morphology and routinely available blood biomarkers, whereas DXA and specialised bone metabolism measurements were confined to the extended model (Figure 1F). Overall, TF90 occurred in 307 patients (18.2%); persistent pain or immobility was the most frequent component (n = 191), followed by secondary displacement (n = 109), FFP-related readmission (n = 95), revision or reoperation (n = 93), delayed conversion to surgery (n = 83) and mortality (n = 8) (Figure 1G). The 579 component occurrences exceeded the number of affected patients because individual patients could experience multiple qualifying events. The development, temporal validation and Centre E cohorts were broadly comparable across core demographic, clinical, imaging and laboratory characteristics; initial operative management was less frequent in temporal validation and Centre E than in development (42.3% and 38.5% versus 49.4%; *p* = 0.003), whereas TF90 incidence did not differ significantly across cohorts (*p* = 0.126) (Supplementary Table S2). Missingness was limited for most core predictors but was greater for DXA T-score (38.4%), β-CTX (32.2%), osteocalcin (29.8%), PINP (29.6%), PTH (21.7%) and 25(OH)D (16.3%); these variables were retained for fold-specific imputation rather than used as complete case eligibility criteria (Supplementary Table S3). Supplementary Table S4 specifies the operational definitions and assessment windows for the primary composite and the progressively more restrictive objective, major clinical event and hard procedural-or-death endpoints. No individual TF90 component showed significant heterogeneity across the three analytical cohorts; primary TF90 incidence was also similar between initially non-operative and operative patients (18.7% versus 17.7%; *p* = 0.572), although revision or reoperation was more frequent after initial operative management (9.4% versus 2.2%; *p* < 0.001), while delayed conversion was applicable only to initially non-operative patients (Supplementary Table S5).

### 3.2. Admission Characteristics Associated with TF90

**Figure 2:**
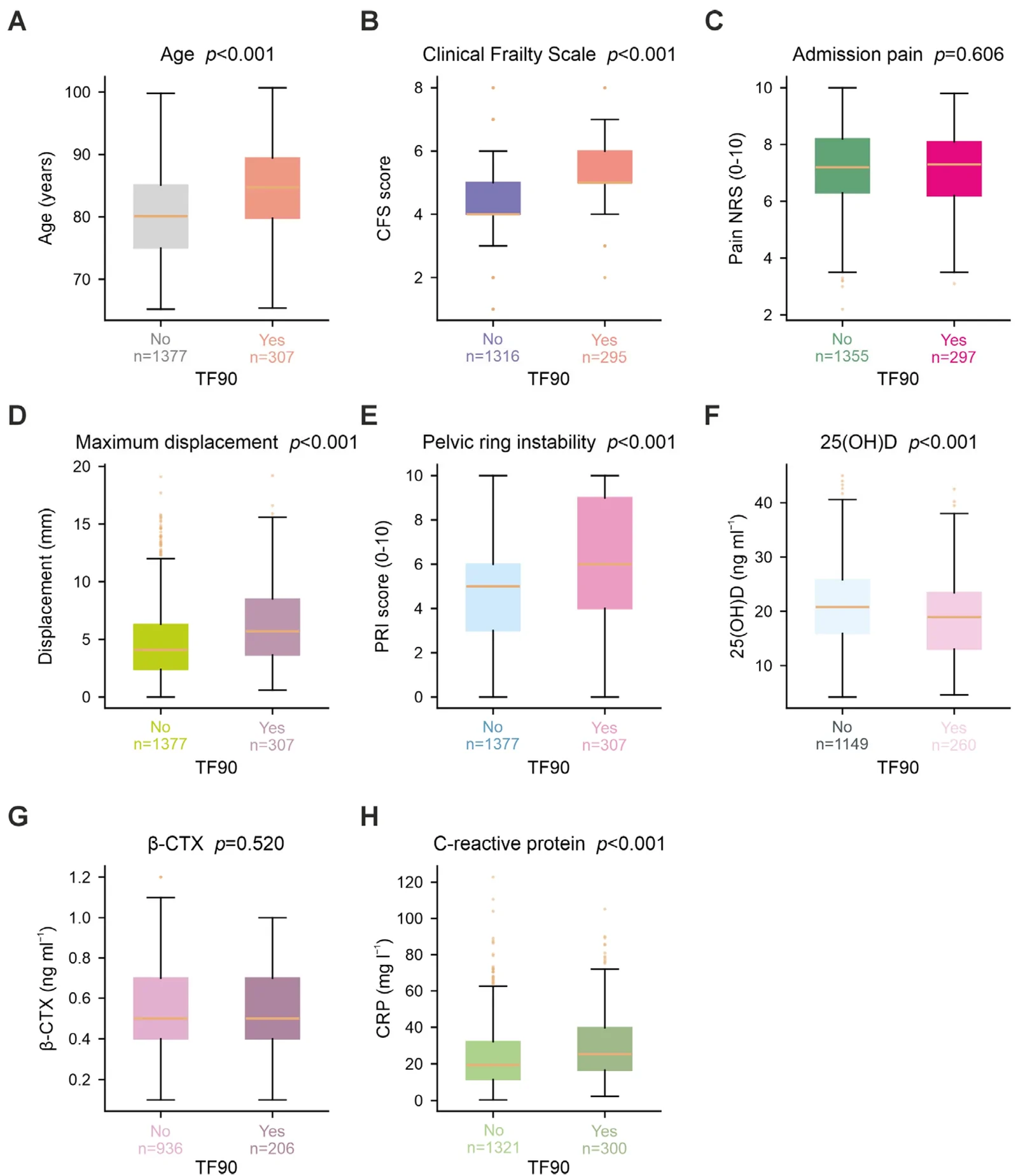
Admission characteristics stratified by TF90 status. (**A**–**H**) Distributions of age (**A**), CFS score (**B**), admission pain NRS score (**C**), maximum fracture displacement (**D**), PRI score (**E**), serum 25(OH)D concentration (**F**), serum β-CTX concentration (**G**) and CRP concentration (**H**) in patients without and with TF90. Boxes indicate the median and interquartile range; whiskers extend to 1.5 times the interquartile range, and observations beyond the whiskers are plotted individually. Numbers beneath each group indicate the available observations for the corresponding variable. *p*-values are two-sided and were calculated using Welch’s *t*-test or the Mann–Whitney U test, as appropriate; no adjustment was made for multiple comparisons.

Patients who developed TF90 were older than those who did not (84.5 versus 80.1 years; *p* < 0.001) (Figure 2A) and had greater prefracture frailty (median CFS, 5 [IQR, 5–6] versus 4 [4–5]; *p* < 0.001) (Figure 2B), whereas admission pain severity was similar between groups (mean NRS, 7.1 versus 7.2; *p* = 0.606) (Figure 2C). Mechanical injury burden was greater among patients with TF90, as reflected by greater maximum fracture displacement (6.3 versus 4.7 mm; *p* < 0.001) (Figure 2D) and higher PRI (6.3 versus 4.7; *p* < 0.001) (Figure 2E). Serum 25(OH)D was lower in the TF90 group (18.7 versus 20.9 ng mL^−1^; *p* < 0.001) (Figure 2F), β-CTX showed no material separation (median, 0.5 ng mL^−1^ in both groups; *p* = 0.520) (Figure 2G), and CRP was higher among patients with TF90 (25.4 [IQR, 16.6–39.8] versus 19.4 [11.5–32.2] mg L^−1^; *p* < 0.001) (Figure 2H). Table 1 further delineated a host-vulnerability phenotype: patients with TF90 had a higher CCI, were more frequently classified as ASA III–V, more commonly resided in assisted-care settings and more often required an assistive device or transfer support before injury; haemoglobin, albumin and eGFR were also lower. The largest clinical standardised differences were observed for CFS (|SMD| = 0.70), age (0.63) and eGFR (0.51), whereas sex, body mass index, known osteoporosis, pre-injury anti-osteoporosis treatment and DXA T-score showed no material differences (Table 1). The distributions of representative continuous variables, including age, body mass index, CCI, CFS, admission pain, DXA T-score, 25(OH)D and haemoglobin, are shown in Supplementary Figure S2A–H; the specialised skeletal variables contained fewer observations than the routinely available core predictors. The categorical case mix is presented in Supplementary Figure S3A–H: women predominated, ASA III was the most frequent class, assisted living was common, most patients were independently ambulant or used a cane or walker before injury, low-energy falls predominated, FFP II and subtype IIb were the largest fracture groups, and conservative management was the most frequent initial strategy. Fracture morphology showed similarly pronounced differences: TF90 was associated with a more severe FFP distribution, greater displacement, higher PRI, posterior ring involvement, bilateral posterior ring involvement, H- or U-shaped sacral morphology, bilateral pubic-ramus fracture, bilateral sacral-ala fracture and acetabular involvement, whereas L5 transverse-process fracture showed little separation (Table 2). Unadjusted TF90 incidence was correspondingly higher across these adverse morphological features, while increasing only modestly across Denis zones (Supplementary Figure S4A–H). Neither the proportion receiving initial operative treatment nor the overall distribution of the six treatment strategies differed significantly by TF90 status (*p* = 0.572 and *p* = 0.252, respectively) (Table 2). PRI construction and scoring are detailed in Supplementary Table S6. In the CFS double-rating analysis, quadratic weighted κ was 0.863, ICC(2,1) was 0.864, exact agreement was 63.1% and agreement within one point was 92.7% (Supplementary Table S7).

### 3.3. Predictive Performance, Calibration and Incremental Model Value

**Figure 3:**
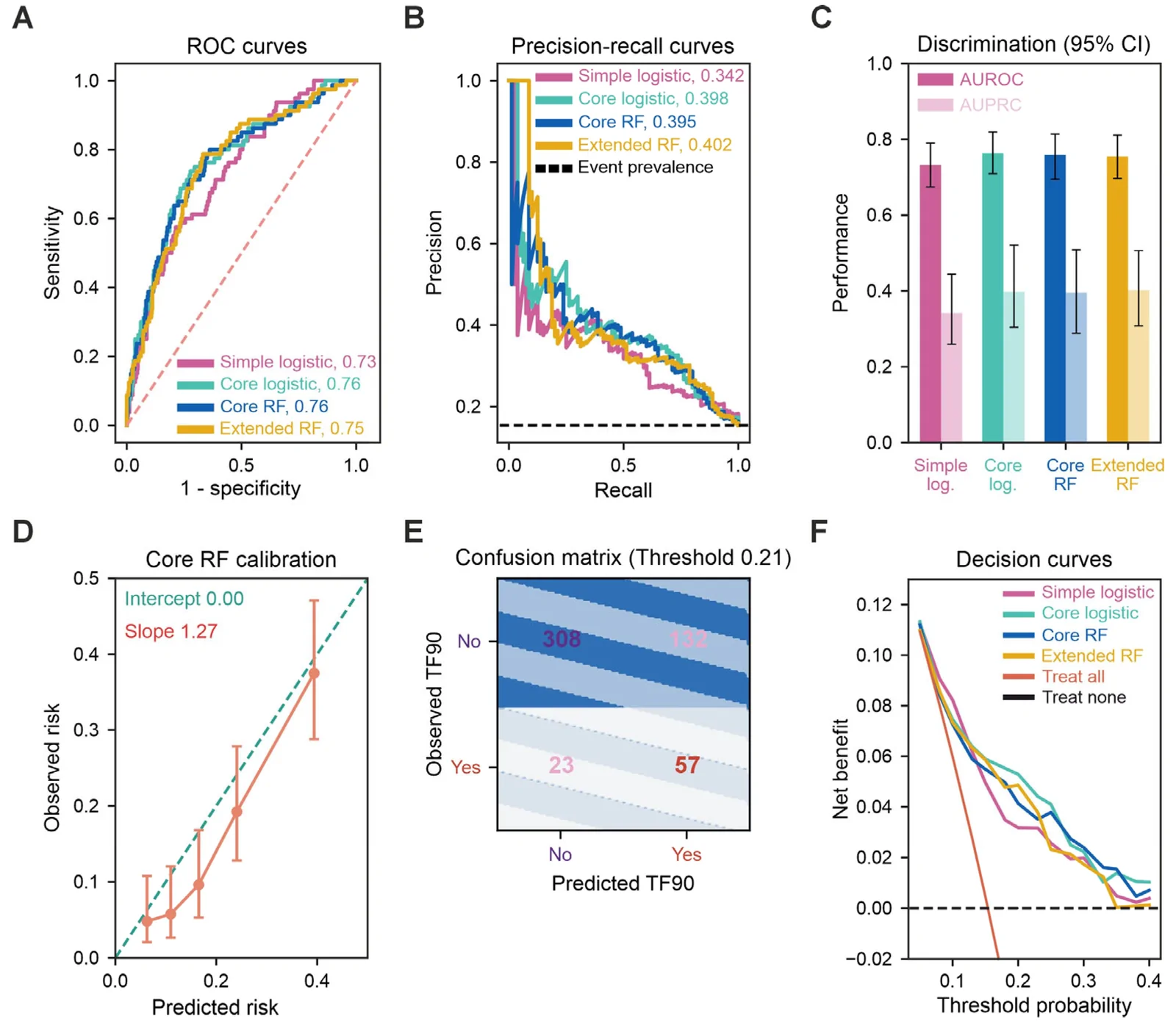
Discrimination, calibration and clinical utility in temporal validation. (**A**) Receiver operating characteristic curves for the simple logistic, core logistic, core random forest (RF) and extended RF models; values in the legend denote AUROC. (**B**) Precision–recall curves for the four candidate models; values in the legend denote AUPRC, and the horizontal dashed line indicates the observed TF90 prevalence. (**C**) AUROC and AUPRC estimates with patient-level bootstrap 95% confidence intervals. (**D**) Calibration of the core RF across grouped predicted-risk intervals. Points represent observed TF90 proportions, vertical bars indicate 95% confidence intervals, and the diagonal dashed line denotes perfect calibration. (**E**) Confusion matrix obtained after applying the development-derived core RF threshold of 0.209 unchanged to the temporal validation cohort, without re-optimisation. (**F**) Decision curves across threshold probabilities of 0.05–0.40, with treat-all and treat-none strategies shown as reference. AUROC, area under the receiver operating characteristic curve; AUPRC, area under the precision–recall curve; TF90, 90-day treatment failure.

All four candidate models showed moderate discrimination in temporal validation, with AUROCs of 0.732 (95% CI, 0.674–0.790) for the simple logistic model, 0.763 (0.709–0.819) for the core logistic model, 0.759 (0.695–0.814) for the core RF and 0.755 (0.697–0.811) for the extended RF (Figure 3A). Precision–recall performance was similarly close, with AUPRCs of 0.342, 0.398, 0.395 and 0.402, respectively, against an event prevalence of 15.4% (Figure 3B); the joint display of AUROC and AUPRC estimates showed substantial overlap in uncertainty and no consistent advantage of greater model complexity (Figure 3C). Core RF calibration was broadly preserved, with an intercept of 0.00 (95% CI, −0.51 to 0.48) and a slope of 1.27 (0.90–1.64) (Figure 3D). Applying the development-derived threshold of 0.209 unchanged to temporal validation yielded 57 true positives, 132 false positives, 308 true negatives and 23 false negatives (Figure 3E). Decision curve analysis indicated positive net benefit over restricted portions of the evaluated 0.05–0.40 threshold range, with limited separation among candidate models (Figure 3F). Table 3 reports discrimination, probabilistic accuracy and calibration across all three analytical settings: development out-of-fold AUROCs ranged from 0.716 to 0.739; temporal validation Brier scores ranged from 0.114 to 0.119; and, in Centre E, AUROCs ranged from 0.720 to 0.751, AUPRCs from 0.338 to 0.379 and Brier scores from 0.145 to 0.153. Centre E calibration estimates were imprecise because of the smaller cohort, and this assessment represented internal–external validation within the participating network rather than independent external validation (Table 3). Relative to the simple logistic model, the core logistic model increased temporal validation AUROC by 0.032 (95% CI, 0.001–0.061; *p* = 0.047) and reduced the Brier score by 0.005 (95% CI, −0.009 to −0.000; *p* = 0.033), whereas its AUPRC increment remained uncertain (ΔAUPRC, 0.056; *p* = 0.077) (Table 4). Differences between the core RF and simple logistic model did not exclude no difference, and the extended RF did not improve consistently upon the core RF; no paired comparison was significant in Centre E (Table 4). Expanded comparisons across AUPRC, Brier score, sensitivity, specificity, PPV, NPV and F1 score likewise demonstrated close performance among the four models (Supplementary Figure S5A–H). Grouped fivefold cross-validation selected C = 0.05 for the simple logistic model and C = 0.005 for the core logistic model; the selected core and extended RF configurations each contained 300 trees with maximum depths of 4 and 6, respectively, a minimum leaf size of 8, square-root feature sampling and balanced subsampling (Supplementary Table S8). The core logistic model achieved the highest mean cross-validated AUPRC (0.405), followed by the simple logistic model (0.401), whereas both RF configurations achieved 0.389 (Supplementary Table S9). Collectively, neither RF modelling nor the addition of specialised skeletal health measurements provided a reproducible validation advantage over the core penalised logistic model.

### 3.4. Model Interpretation and Development-Derived Risk Stratification

**Figure 4:**
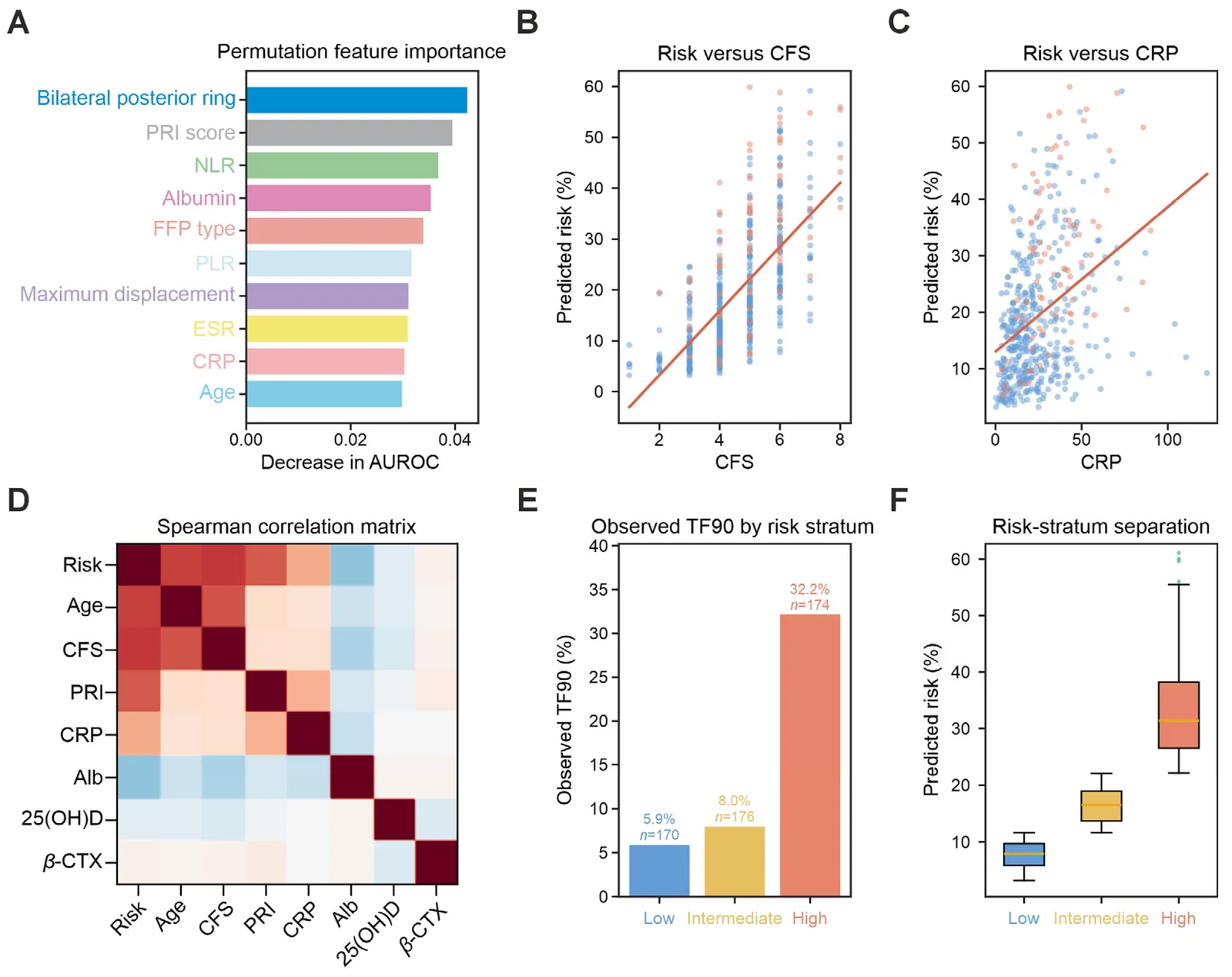
Model interpretation and development-derived risk stratification. (**A**) Permutation importance of the ten highest-ranking predictors in the core random forest (RF), quantified as the mean decrease in validation AUROC after permutation of each variable. (**B,C**) Patient-level core RF predicted risk according to CFS (**B**) and CRP (**C**) in the temporal validation cohort. Colours indicate observed TF90 status, and fitted lines depict descriptive trends. (**D**) Spearman correlation matrix showing associations between predicted risk and selected clinical, mechanical and biochemical variables. Red indicates positive correlations and blue indicates negative correlations, with darker shades representing stronger correlations and near-white cells indicating correlations close to zero. (**E**) Observed TF90 incidence across development-derived low-, intermediate- and high-risk strata in temporal validation. Risk strata were defined using fixed cut-offs of <0.116, 0.116 to <0.221 and ≥0.221, comprising 170, 176 and 174 patients, respectively. (**F**) Distribution of predicted risk across the three strata. Boxes indicate the median and interquartile range; whiskers extend to 1.5 times the interquartile range, and observations beyond the whiskers are plotted individually. AUROC, area under the receiver operating characteristic curve; CFS, Clinical Frailty Scale; CRP, C-reactive protein; TF90, 90-day treatment failure.

Permutation analysis identified a distributed, multidomain prediction architecture rather than dependence on a single dominant variable (Figure 4A). The largest decreases in validation AUROC followed permutation of bilateral posterior ring involvement (0.0423), PRI (0.0395), NLR (0.0368), albumin (0.0353), FFP category (0.0339), PLR (0.0317), maximum displacement (0.0311), ESR (0.0309), CRP (0.0303) and age (0.0298); the complete ranking, including FFP subtype, D-dimer, sodium, lymphocyte count and sacral fracture zone at ranks 11–15, is reported in Supplementary Table S10. Because several morphological predictors were correlated, these estimates quantified model dependence and could be shared or redistributed among related variables; they were not interpreted as independent or causal effects. Predicted risk increased across the observed range of CFS (Figure 4B) and CRP (Figure 4C), although substantial patient-level overlap remained. The correlation matrix showed positive associations of predicted risk with age, CFS, PRI and CRP and an inverse association with albumin (Figure 4D). The broader pairwise structure was clinically coherent: age correlated with CFS (ρ = 0.63), PRI correlated strongly with maximum displacement (ρ = 0.83), predicted risk correlated with PRI (ρ = 0.62), and both CFS and CRP correlated inversely with albumin (ρ = −0.32 and −0.23, respectively); 25(OH)D correlated only weakly with DXA T-score (ρ = −0.06) (Supplementary Figure S6A–H). Mean predicted risk increased across higher grouped values of age, CFS, maximum displacement, PRI and CRP, whereas admission pain, 25(OH)D and β-CTX showed non-monotonic gradients and were therefore interpreted as descriptive characteristics of the fitted prediction surface rather than adjusted effects (Supplementary Figure S7A–H). When development-derived cut-offs of <0.116, 0.116 to <0.221 and ≥0.221 were applied unchanged to temporal validation, 170 patients were classified as low risk, 176 as intermediate risk and 174 as high risk. TF90 occurred in 10 low-risk patients (5.9%), 14 intermediate-risk patients (8.0%) and 56 high-risk patients (32.2%) (Figure 4E). The high-risk stratum contained 70.0% of all temporal validation events, and predicted probabilities were clearly separated across the three strata (Figure 4F). The limited difference between the low- and intermediate-risk event rates indicated that the principal stratification signal was enrichment of a high-risk subgroup rather than uniform ordinal separation across all three categories.

### 3.5. Event Timing, Treatment-Stratified Patterns and Threshold Trade-Offs

**Figure 5:**
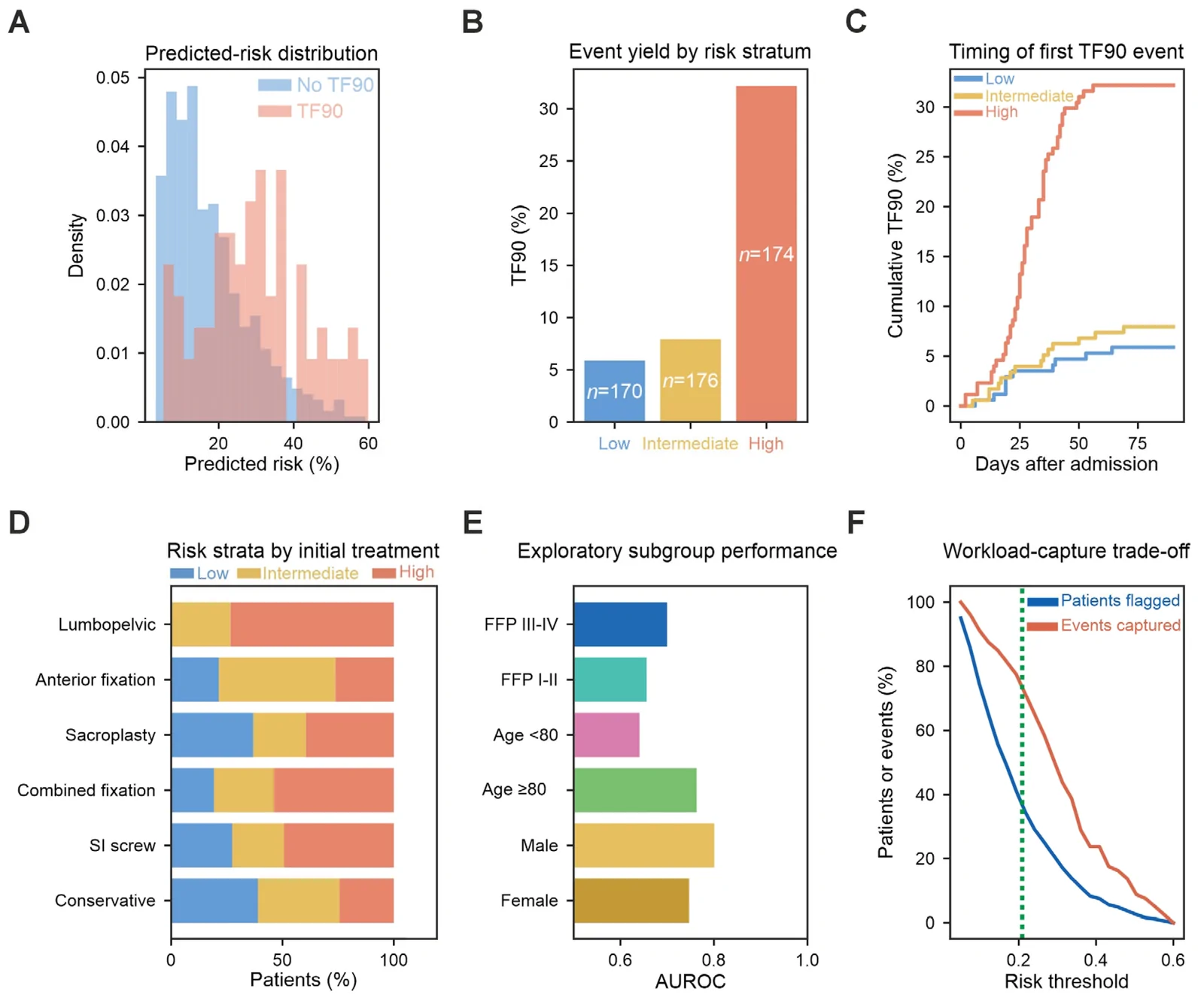
Clinical risk stratification, event timing and threshold-dependent trade-offs. (**A**) Distribution of core random forest (RF) predicted risk according to observed TF90 status. (**B**) Observed TF90 incidence across the development-derived low-, intermediate- and high-risk strata. (**C**) Unadjusted cumulative proportion of patients experiencing a first TF90 component within 90 days after admission, stratified by risk group. (**D**) Distribution of risk strata within each initial treatment category. These treatment-stratified patterns reflect differences in observed case mix and should not be interpreted as comparative treatment effects. (**E**) Exploratory subgroup AUROC estimates according to sex, age and grouped FFP category. No formal interaction testing was performed. (**F**) Proportions of patients classified as positive and TF90 events captured across candidate risk thresholds. The vertical dotted line indicates the development-derived core RF threshold of 0.209. AUROC, area under the receiver operating characteristic curve; FFP, fragility fractures of the pelvis; TF90, 90-day treatment failure.

Predicted-risk distributions were shifted towards higher values among patients who developed TF90, although considerable overlap remained between event and non-event groups (Figure 5A). Observed event rates reproduced the development-derived risk-stratum pattern, with TF90 occurring in 5.9%, 8.0% and 32.2% of the low-, intermediate- and high-risk groups, respectively (Figure 5B). The cumulative proportion experiencing a first TF90 event separated primarily during the first 50 days after admission: the high-risk curve rose rapidly before approaching a plateau near 32%, whereas both lower-risk groups remained below 10% throughout 90 days (Figure 5C). These unadjusted cumulative proportions did not account for competing events or post-landmark treatment changes. Risk-stratum composition differed across initial treatment categories, with high-risk classifications most frequent among patients undergoing lumbopelvic fixation or combined anterior–posterior fixation and least frequent among those managed conservatively; conversely, low-risk classifications were most common in the conservative treatment group (Figure 5D). These distributions reflected fracture severity, frailty and treatment selection mechanisms and were not interpreted as comparative treatment effects. Exploratory subgroup discrimination ranged from approximately 0.64 to 0.80, with AUROCs of 0.75 in women, 0.80 in men, 0.76 in patients aged ≥80 years and 0.64 in those aged <80 years (Figure 5E). More granular subgroup analyses showed AUROCs of 0.52, 0.67, 0.68 and 0.66 across FFP I–IV, respectively; the FFP I estimate was based on only four events and was therefore highly uncertain (Supplementary Figure S8A–H). These exploratory analyses were not powered for formal interaction testing. At the fixed threshold of 0.209, 189 of 520 patients (36.3%) were classified as positive, capturing 57 of 80 TF90 events; sensitivity was 71.3%, specificity 70.0%, PPV 30.2%, NPV 93.1% and F1 score 42.4% (Figure 5F). Threshold-dependent profiles demonstrated the expected trade-off between clinical workload and event capture: increasing the threshold improved specificity and PPV but reduced sensitivity, NPV, F1 score after its local maximum, the proportion of patients flagged and the proportion of TF90 events captured; the fixed threshold of 0.209 lay in a region retaining 71.3% sensitivity and positive net benefit (Supplementary Figure S9A–H). These operating characteristics support surveillance enrichment and structured reassessment.

### 3.6. Internal–External Transportability, Calibration Heterogeneity and Sensitivity Analyses

**Figure 6:**
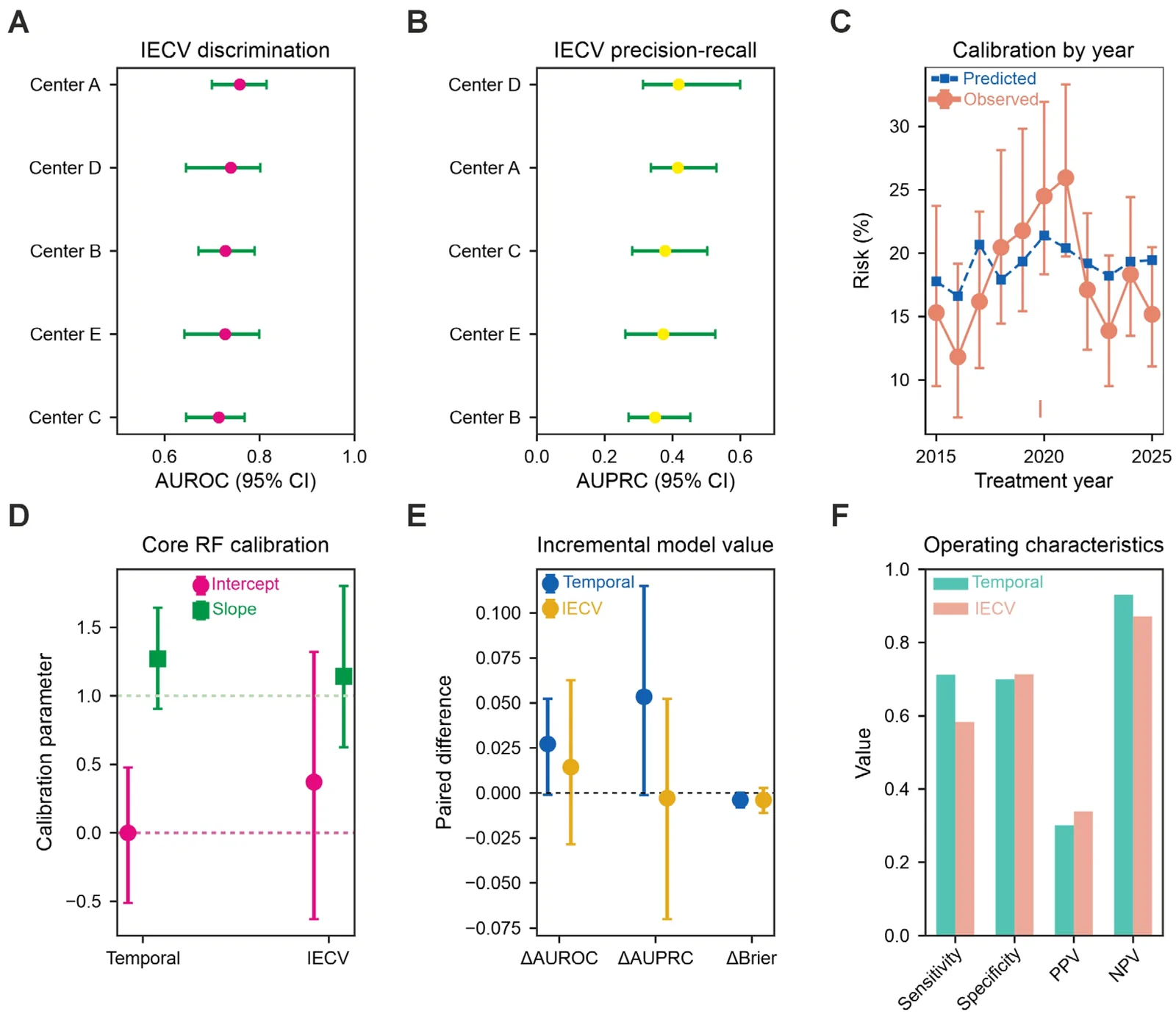
Internal–external transportability, calibration heterogeneity and incremental model performance. (**A**,**B**) Performance of the core random forest (RF) in leave-one-centre-out internal–external cross-validation (IECV), expressed as AUROC (**A**) and AUPRC (**B**) with 95% confidence intervals. These analyses assess transportability across the participating centres and do not constitute independent external validation. (**C**) Mean predicted risk and observed TF90 incidence according to treatment year. Vertical bars indicate 95% confidence intervals for the observed incidence. (**D**) Calibration intercepts and slopes, with 95% confidence intervals, in the temporal validation cohort and the fixed held-out Centre E internal–external validation cohort. Reference values are 0 for the calibration intercept and 1 for the calibration slope. (**E**) Paired differences in AUROC, AUPRC and Brier score between the core RF and simple logistic model in temporal validation and Centre E validation. Positive differences in AUROC and AUPRC and negative differences in Brier score favour the core RF. (**F**) Sensitivity, specificity, PPV and NPV after applying the development-derived core RF threshold of 0.209 unchanged to both validation cohorts. AUROC, area under the receiver operating characteristic curve; AUPRC, area under the precision–recall curve; NPV, negative predictive value; PPV, positive predictive value; TF90, 90-day treatment failure.

Leave-one-centre-out IECV showed moderate discrimination across Centres A–E, with AUROCs of 0.758, 0.728, 0.714, 0.739 and 0.727, respectively (Figure 6A), and corresponding AUPRCs of 0.416, 0.349, 0.379, 0.418 and 0.373 (Figure 6B). Observed and predicted annual risks followed broadly similar trajectories, although TF90 risk was underestimated in 2020 and 2021, when observed incidences were 24.5% and 25.9% compared with mean predicted risks of 21.4% and 20.4%, respectively (Figure 6C). For the fixed core RF, the calibration intercept and slope were 0.00 and 1.27 in temporal validation and 0.37 and 1.14 in Centre E (Figure 6D). Relative to the simple logistic model, the core RF showed only small and statistically uncertain differences in AUROC, AUPRC and Brier score in both validation settings (Figure 6E). At the development-derived threshold of 0.209, Centre E showed lower sensitivity than temporal validation, with broadly similar specificity, modest PPV and high NPV (Figure 6F). Complete centre-specific IECV estimates are reported in Table 5. Across centres, Brier scores ranged from 0.126 to 0.148, calibration intercepts from −0.21 to 0.26 and calibration slopes from 0.85 to 1.34. The leave-one-centre-out Centre E AUROC of 0.727 differed from the fixed held-out Centre E AUROC of 0.745 because these estimates arose from distinct validation procedures (Table 5). The core RF retained moderate discrimination when TF90 was restricted to progressively more objective or clinically severe outcomes (Table 6). In temporal validation, AUROC/AUPRC/Brier values were 0.759/0.395/0.115 for primary TF90, 0.735/0.332/0.108 for the objective composite, 0.742/0.271/0.090 for the major clinical event endpoint and 0.772/0.277/0.076 for the hard procedural-or-death endpoint. Corresponding Centre E AUROCs were 0.745, 0.739, 0.699 and 0.711, with increasing uncertainty as event counts declined (Table 6). Model performance was also examined under alternative representations of pelvic ring mechanical instability. In this sensitivity analysis, the composite PRI score was compared with a component-based representation in which FFP type, posterior ring involvement, bilateral posterior ring involvement, maximum fracture displacement and H- or U-shaped sacral morphology were entered separately, while all other predictors and RF specifications were held constant. ROC curves were closely aligned in both temporal validation and Centre E, and precision–recall performance showed no consistent advantage for either representation (Supplementary Figure S1). These findings indicate that model performance was broadly preserved when the composite PRI score was replaced by its constituent morphological features, supporting the robustness of prediction to the representation of underlying mechanical instability information rather than to a particular composite weighting scheme. Calibration nevertheless varied across centres, treatment years and initial treatment strata. Centre-specific O ratios ranged from 0.88 to 1.14, year-specific O ratios from 0.71 to 1.27 and calibration slopes from 0.60 to 1.44. Initially non-operative and operative patients had O ratios of 1.13 and 0.79 and calibration slopes of 1.50 and 0.86, respectively (Supplementary Table S11). These patterns described heterogeneity under observed care and were not interpreted as treatment effects. Component-specific discrimination was highest for revision or reoperation in temporal validation (AUROC, 0.832; 95% CI, 0.748–0.902; 27 events), followed by persistent pain or immobility (0.730), secondary displacement (0.729), delayed conversion (0.713) and FFP-related readmission (0.683). Estimates in Centre E were less stable, particularly for delayed conversion, and mortality was not modelled separately because only eight deaths occurred (Supplementary Table S12). Collectively, these analyses supported moderate within-network transportability while identifying expected heterogeneity in calibration and uncertainty in less frequent component outcomes.

## 4. Discussion

This multicentre study shows that early failure after FFP is best understood as the consequence of an interaction between pelvic ring instability and host vulnerability rather than as an anatomical problem alone. TF90 occurred in almost one in five patients and increased markedly across FFP categories, yet fracture severity did not fully account for outcome heterogeneity. Older age, greater frailty, impaired prefracture mobility, renal dysfunction, hypoalbuminaemia, systemic inflammation, bilateral posterior ring injury and greater displacement collectively characterised patients at higher risk. By combining these domains at a strictly defined pretreatment landmark, the models achieved moderate discrimination in temporal and held-out-centre validation. The principal clinical signal was not uniform separation across all risk levels, but identification of a high-risk subgroup with a TF90 incidence of 32.2% that contained 70.0% of all events in temporal validation.

These findings reinforce the concept of FFP as a geriatric fracture syndrome. Conventional assessment appropriately prioritises CT-defined morphology and posterior ring integrity, but anatomically similar injuries may follow different trajectories because patients differ substantially in physiological reserve and capacity for rehabilitation [12,16]. The strong contribution of CFS, age, albumin, inflammatory indices and renal function indicates that the consequences of mechanical instability are conditioned by systemic resilience. Conversely, frailty alone cannot replace anatomical assessment: bilateral posterior ring involvement, PRI, displacement and FFP category remained among the most influential predictors. The observed performance therefore appears to derive from integration of complementary rather than interchangeable information. PRI offered a compact representation of mechanical instability, but it is study-specific and correlated with several constituent imaging features; its predictive importance should not be interpreted as evidence of independent biological or causal effects [16,45,46].

The comparison between modelling strategies is clinically important. The core logistic model modestly improved temporal validation discrimination and probabilistic accuracy relative to the parsimonious benchmark, whereas the core RF did not provide a statistically robust advantage. The extended RF, despite incorporating DXA and specialised bone metabolism measurements, offered no reproducible improvement over models based on routine admission information. This absence of incremental value is informative rather than negative. DXA and bone turnover markers may remain relevant to long-term skeletal health and secondary fracture prevention, but their acute prognostic contribution appears limited once age, frailty, fracture morphology, inflammation, nutrition and renal function are considered [33,36,47]. Their higher missingness and inconsistent availability also reduce operational value at admission. More broadly, the results caution against assuming that algorithmic complexity necessarily improves clinical prediction. In this setting, a transparent penalised model performed at least as well as a more flexible ensemble method.

The primary endpoint was deliberately broader than mortality or reoperation alone because early failure after FFP frequently manifests through persistent pain, inability to mobilise, progressive displacement, delayed surgery or unplanned readmission [10,42]. These events are clinically meaningful, but they are not equivalent in severity or objectivity. We therefore reported each component separately and repeated the analysis using progressively more restrictive endpoints. Discrimination remained moderate for the objective, major event and hard procedural-or-death composites, supporting the robustness of the overall risk signal. Nevertheless, the composite should be interpreted as a pragmatic summary of failure pathways rather than as a homogeneous biological outcome. Persistent pain and mobility failure may be influenced by rehabilitation intensity, analgesic practice, social support and discharge pathways, whereas reoperation and delayed conversion are partly shaped by local treatment thresholds. Component-specific estimates, particularly in Centre E, were consequently imprecise when event counts were small.

The fixed threshold of 0.209 illustrates the potential and the limitations of clinical deployment. In temporal validation, it achieved a sensitivity of 71.3%, specificity of 70.0% and NPV of 93.1%, while classifying 36.3% of patients as positive. These operating characteristics may be useful for surveillance enrichment: patients above the threshold could undergo earlier multidisciplinary review, predefined mobilisation assessment, closer pain monitoring or repeat imaging when clinically indicated. The modest PPV of 30.2%, however, means that most flagged patients would not experience TF90. The model should therefore not trigger automatic operative treatment, prolonged admission or resource-intensive intervention in isolation. Decision curve analysis suggested potential net benefit only across restricted threshold ranges, and no clinical impact study was performed [48,49]. Whether model-guided monitoring improves outcomes, reduces delayed deterioration or remains cost-effective must be tested prospectively.

Transportability was encouraging. Leave-one-centre-out IECV produced broadly similar discrimination across the five hospitals, and the fixed Centre E evaluation supported performance in a fully held-out participating centre. These analyses provide stronger evidence than a random split because they challenge the model across time and institutional boundaries. They do not, however, constitute independent external validation. Calibration varied by centre, treatment year and initial management stratum, with underprediction during years of higher observed TF90 incidence. Such variation may reflect changes in case mix, admission pathways, surgical practice, rehabilitation capacity or follow-up ascertainment. The lower discrimination observed in some subgroups and the wide confidence intervals for calibration further indicate that local recalibration may be required before implementation. The treatment-stratified findings must also remain descriptive because treatment allocation was not random and was strongly influenced by fracture severity, frailty and clinician judgement.

Several limitations warrant emphasis. The retrospective design creates unavoidable dependence on the completeness and consistency of routinely collected data. Although the prediction landmark and nested preprocessing reduced temporal and analytical leakage, unmeasured differences in imaging interpretation, mobilisation assessment and follow-up may remain. Missingness was substantial for DXA and specialised skeletal biomarkers, and imputation cannot fully recover information that was never measured. The number of deaths was too small to support a separate mortality model, while subgroup and component-specific analyses were underpowered for formal interaction testing. The composite endpoint may remain sensitive to institutional practice despite prespecified definitions and blinded adjudication. In addition, permutation importance and predicted-risk gradients describe the fitted model rather than causal relationships. Finally, risk thresholds and strata were derived within the development cohort and require confirmation outside the participating network.

## 5. Conclusions

In conclusion, admission-based multimodal prediction can identify older patients with FFP who are more likely to experience treatment failure within 90 days. The clinically relevant information lies in the joint assessment of pelvic ring mechanics, frailty and systemic vulnerability, whereas specialised skeletal measurements and greater algorithmic complexity added little reproducible value. The model is best positioned as a tool for prognostic enrichment and structured reassessment, not as an automated determinant of surgery. Independent prospective external validation, local recalibration and clinical impact evaluation are required before routine use.

## Figures and Tables

**Table 1:** Baseline clinical and laboratory characteristics according to 90-day treatment failure status.

Characteristic	Overall (n = 1684)	No TF90 (n = 1377)	TF90 (n = 307)	|SMD|	*p*
Age, years	80.9 (7.3)	80.1 (7.1)	84.5 (6.9)	0.63	<0.001
Female sex	1235 (73.3)	1011 (73.4)	224 (73.0)	0.01	0.870
Body mass index, kg m^−2^	23.2 (3.3)	23.2 (3.2)	23.5 (3.4)	0.08	0.235
Charlson Comorbidity Index	2.0 [1.0–4.0]	2.0 [1.0–3.0]	3.0 [2.0–4.0]	0.46	<0.001
Clinical Frailty Scale	5.0 [4.0–6.0]	4.0 [4.0–5.0]	5.0 [5.0–6.0]	0.70	<0.001
ASA class III–V	1236 (73.4)	991 (72.0)	245 (79.8)	0.18	0.005
Assisted living or nursing home	1006 (59.7)	800 (58.1)	206 (67.1)	0.19	0.004
Cane/walker or wheelchair/bed-to-chair before injury	790 (46.9)	616 (44.7)	174 (56.7)	0.24	<0.001
Admission pain NRS	7.1 (1.4)	7.2 (1.4)	7.1 (1.4)	0.05	0.606
Haemoglobin, g L^−1^	119.7 (15.9)	120.2 (15.8)	117.5 (16.0)	0.17	0.008
C-reactive protein, mg L^−1^	20.8 [12.4–33.8]	19.4 [11.5–32.2]	25.4 [16.6–39.8]	0.38	<0.001
Albumin, g L^−1^	36.1 (3.8)	36.4 (3.8)	34.9 (3.6)	0.38	<0.001
eGFR, mL min^−1^ 1.73 m^−2^	68.7 (15.7)	70.1 (15.6)	62.3 (14.8)	0.51	<0.001
Known osteoporosis	923 (54.8)	742 (53.9)	181 (59.0)	0.10	0.106
Pre-injury anti-osteoporosis pharmacotherapy	245 (14.5)	192 (13.9)	53 (17.3)	0.09	0.136
DXA lumbar or hip T-score	−3.0 (0.7)	−3.0 (0.7)	−3.0 (0.7)	0.08	0.322
25-hydroxyvitamin D, ng mL^−1^	20.5 (7.2)	20.9 (7.1)	18.7 (7.4)	0.29	<0.001
β-CTX, ng mL^−1^	0.5 [0.4–0.7]	0.5 [0.4–0.7]	0.5 [0.4–0.7]	0.03	0.520

**Table 2:** Fracture morphology and initial treatment according to 90-day treatment failure status.

Characteristic	Overall (n = 1684)	No TF90 (n = 1377)	TF90 (n = 307)	|SMD|	*p*
Maximum fracture displacement, mm	5.0 (3.2)	4.7 (3.1)	6.3 (3.5)	0.47	<0.001
Pelvic Ring Instability score (0–10)	5.0 (2.8)	4.7 (2.7)	6.3 (2.6)	0.61	<0.001
FFP category				0.44	<0.001
I	297 (17.6)	273 (19.8)	24 (7.8)		
II	808 (48.0)	702 (51.0)	106 (34.5)		
III	355 (21.1)	261 (19.0)	94 (30.6)		
IV	224 (13.3)	141 (10.2)	83 (27.0)		
Posterior ring involvement	1387 (82.4)	1104 (80.2)	283 (92.2)	0.35	<0.001
Bilateral posterior ring involvement	597 (35.5)	430 (31.2)	167 (54.4)	0.48	<0.001
H- or U-shaped sacral fracture	110 (6.5)	73 (5.3)	37 (12.1)	0.24	<0.001
Any pubic ramus fracture	1610 (95.6)	1308 (95.0)	302 (98.4)	0.19	0.009
Bilateral pubic ramus fracture	765 (45.4)	586 (42.6)	179 (58.3)	0.32	<0.001
Any sacral ala fracture	1387 (82.4)	1104 (80.2)	283 (92.2)	0.35	<0.001
Bilateral sacral ala fracture	661 (39.3)	501 (36.4)	160 (52.1)	0.32	<0.001
Acetabular involvement	117 (6.9)	84 (6.1)	33 (10.7)	0.17	0.004
L5 transverse-process fracture	231 (13.7)	186 (13.5)	45 (14.7)	0.03	0.596
Initial operative treatment	776 (46.1)	639 (46.4)	137 (44.6)	0.04	0.572
Initial treatment strategy				0.12	0.252
Conservative	908 (53.9)	738 (53.6)	170 (55.4)		
Percutaneous SI screw	249 (14.8)	214 (15.5)	35 (11.4)		
Sacroplasty	149 (8.8)	125 (9.1)	24 (7.8)		
Anterior ring fixation	148 (8.8)	122 (8.9)	26 (8.5)		
Combined anterior-posterior fixation	160 (9.5)	123 (8.9)	37 (12.1)		
Lumbopelvic fixation	70 (4.2)	55 (4.0)	15 (4.9)		

**Table 3:** Predictive performance of candidate models across development and validation cohorts.

Model	Cohort	N	Events, n (%)	AUROC (95% CI)	AUPRC (95% CI)	Brier Score (95% CI)	Calibration Intercept (95% CI)	Calibration Slope (95% CI)
Simple logistic	Development OOF	985	191 (19.4)	0.739 (0.703–0.782)	0.381 (0.328–0.456)	0.139 (0.126–0.153)	0.00 (−0.30–0.35)	1.00 (0.80–1.23)
Simple logistic	Temporal validation	520	80 (15.4)	0.732 (0.674–0.790)	0.342 (0.260–0.444)	0.119 (0.104–0.137)	−0.20 (−0.68–0.29)	1.04 (0.76–1.41)
Simple logistic	Internal–external	179	36 (20.1)	0.731 (0.639–0.816)	0.378 (0.259–0.558)	0.149 (0.110–0.187)	0.36 (−0.57–1.54)	1.03 (0.54–1.68)
Core logistic	Development OOF	985	191 (19.4)	0.734 (0.693–0.768)	0.381 (0.324–0.455)	0.140 (0.127–0.155)	0.00 (−0.28–0.34)	1.00 (0.81–1.21)
Core logistic	Temporal validation	520	80 (15.4)	0.763 (0.709–0.819)	0.398 (0.304–0.521)	0.114 (0.095–0.129)	−0.07 (−0.46–0.41)	1.17 (0.89–1.53)
Core logistic	Internal–external	179	36 (20.1)	0.720 (0.635–0.804)	0.338 (0.243–0.502)	0.153 (0.115–0.190)	0.18 (−0.57–1.19)	0.92 (0.50–1.48)
Core RF	Development OOF	985	191 (19.4)	0.716 (0.675–0.757)	0.370 (0.310–0.440)	0.141 (0.127–0.154)	0.00 (−0.33–0.31)	1.00 (0.79–1.24)
Core RF	Temporal validation	520	80 (15.4)	0.759 (0.695–0.814)	0.395 (0.288–0.508)	0.115 (0.099–0.132)	0.00 (−0.51–0.48)	1.27 (0.90–1.64)
Core RF	Internal–external	179	36 (20.1)	0.745 (0.648–0.830)	0.375 (0.251–0.535)	0.145 (0.105–0.183)	0.37 (−0.63–1.32)	1.14 (0.62–1.80)
Extended RF	Development OOF	985	191 (19.4)	0.724 (0.686–0.762)	0.369 (0.306–0.446)	0.141 (0.127–0.155)	0.00 (−0.31–0.33)	1.00 (0.81–1.24)
Extended RF	Temporal validation	520	80 (15.4)	0.755 (0.697–0.811)	0.402 (0.308–0.506)	0.116 (0.099–0.131)	−0.05 (−0.51–0.45)	1.22 (0.89–1.65)
Extended RF	Internal–external	179	36 (20.1)	0.751 (0.666–0.826)	0.379 (0.278–0.562)	0.145 (0.114–0.180)	0.33 (−0.44–1.40)	1.12 (0.66–1.74)

**Table 4:** Incremental predictive performance relative to simpler reference models.

Cohort	Comparison	ΔAUROC (95% CI)	*p*	ΔAUPRC (95% CI)	*p*	ΔBrier (95% CI)	*p*
Temporal validation	Core logistic vs. Simple logistic	+0.032 (+0.001 to +0.061)	0.047	+0.056 (−0.002 to +0.112)	0.077	−0.005 (−0.009 to −0.000)	0.033
Temporal validation	Core RF vs. Simple logistic	+0.027 (−0.001 to +0.052)	0.060	+0.053 (−0.001 to +0.115)	0.060	−0.004 (−0.008 to +0.000)	0.070
Temporal validation	Extended RF vs. Core RF	−0.004 (−0.020 to +0.011)	0.636	+0.007 (−0.048 to +0.045)	0.885	+0.001 (−0.001 to +0.003)	0.369
Internal–external	Core logistic vs. Simple logistic	−0.011 (−0.057 to +0.032)	0.639	−0.040 (−0.110 to +0.012)	0.136	+0.004 (−0.002 to +0.011)	0.246
Internal–external	Core RF vs. Simple logistic	+0.014 (−0.029 to +0.063)	0.552	−0.003 (−0.070 to +0.052)	0.895	−0.004 (−0.011 to +0.003)	0.270
Internal–external	Extended RF vs. Core RF	+0.006 (−0.017 to +0.033)	0.566	+0.003 (−0.026 to +0.033)	0.855	+0.000 (−0.003 to +0.003)	0.968

**Table 5:** Internal–external cross-validation performance of the Core RF model by held-out centre.

Held-Out Centre	N	Events, n (%)	AUROC (95% CI)	AUPRC (95% CI)	Brier Score (95% CI)	Calibration Intercept (95% CI)	Calibration Slope (95% CI)
Centre A	497	88 (17.7)	0.758 (0.700–0.815)	0.416 (0.336–0.530)	0.126 (0.108–0.144)	0.22 (−0.32–0.68)	1.34 (0.99–1.78)
Centre B	405	71 (17.5)	0.728 (0.671–0.789)	0.349 (0.270–0.452)	0.132 (0.111–0.151)	−0.09 (−0.51–0.38)	1.01 (0.75–1.38)
Centre C	335	62 (18.5)	0.714 (0.645–0.768)	0.379 (0.281–0.503)	0.138 (0.116–0.165)	−0.21 (−0.70–0.31)	0.85 (0.57–1.13)
Centre D	268	50 (18.7)	0.739 (0.645–0.801)	0.418 (0.313–0.599)	0.132 (0.106–0.159)	0.17 (−0.55–0.82)	1.13 (0.69–1.58)
Centre E	179	36 (20.1)	0.727 (0.642–0.799)	0.373 (0.261–0.526)	0.148 (0.108–0.183)	0.26 (−0.50–1.02)	0.98 (0.57–1.40)

**Table 6:** Core RF model performance for the primary and sensitivity analysis endpoints.

Endpoint	Cohort	N	Events, n (%)	AUROC (95% CI)	AUPRC (95% CI)	Brier Score (95% CI)	Calibration Slope (95% CI)
Primary TF90 (any of six)	Development OOF	985	191 (19.4)	0.716 (0.675–0.757)	0.370 (0.310–0.440)	0.141 (0.127–0.154)	1.00 (0.79–1.24)
Primary TF90 (any of six)	Temporal validation	520	80 (15.4)	0.759 (0.695–0.814)	0.395 (0.288–0.508)	0.115 (0.099–0.132)	1.27 (0.90–1.64)
Primary TF90 (any of six)	Internal–external	179	36 (20.1)	0.745 (0.648–0.830)	0.375 (0.251–0.535)	0.145 (0.105–0.183)	1.14 (0.62–1.80)
Objective composite	Development OOF	985	156 (15.8)	0.702 (0.657–0.741)	0.268 (0.220–0.331)	0.125 (0.112–0.142)	1.00 (0.74–1.26)
Objective composite	Temporal validation	520	71 (13.7)	0.735 (0.667–0.791)	0.332 (0.243–0.444)	0.108 (0.095–0.125)	1.26 (0.85–1.70)
Objective composite	Internal–external	179	26 (14.5)	0.739 (0.657–0.810)	0.273 (0.180–0.408)	0.115 (0.088–0.147)	1.25 (0.74–1.84)
Major clinical event	Development OOF	985	138 (14.0)	0.682 (0.631–0.726)	0.230 (0.193–0.288)	0.115 (0.100–0.131)	1.00 (0.71–1.29)
Major clinical event	Temporal validation	520	56 (10.8)	0.742 (0.679–0.801)	0.271 (0.192–0.392)	0.090 (0.075–0.108)	1.48 (1.01–2.01)
Major clinical event	Internal–external	179	17 (9.5)	0.699 (0.589–0.822)	0.184 (0.101–0.369)	0.085 (0.059–0.117)	1.16 (0.55–2.31)
Hard procedural/death endpoint	Development OOF	985	104 (10.6)	0.683 (0.637–0.727)	0.170 (0.135–0.221)	0.091 (0.077–0.103)	1.00 (0.71–1.28)
Hard procedural/death endpoint	Temporal validation	520	47 (9.0)	0.772 (0.700–0.836)	0.277 (0.184–0.401)	0.076 (0.061–0.095)	1.73 (1.13–2.46)
Hard procedural/death endpoint	Internal–external	179	13 (7.3)	0.711 (0.570–0.850)	0.162 (0.080–0.358)	0.066 (0.039–0.092)	1.25 (0.49–2.80)

## Data Availability

The datasets used and/or analysed during the current study are available from the corresponding authors upon reasonable request.

## Supplementary Materials

The following supporting information can be downloaded at: https://www.mdpi.com/article/10.3390/jcm15166487/s1.

## Abbreviations

25(OH)D	25-hydroxyvitamin D
β-CTX	β-isomerised C-terminal telopeptide of type I collagen
ASA	American Society of Anesthesiologists
AUPRC	area under the precision–recall curve
AUROC	area under the receiver operating characteristic curve
CCI	Charlson Comorbidity Index
CFS	Clinical Frailty Scale
CI	confidence interval
CRP	C-reactive protein
CT	computed tomography
DCA	decision curve analysis
DXA	dual-energy X-ray absorptiometry
eGFR	estimated glomerular filtration rate
ESR	erythrocyte sedimentation rate
FFP	fragility fractures of the pelvis
ICC	intraclass correlation coefficient
IECV	internal–external cross-validation
IQR	interquartile range
NLR	neutrophil-to-lymphocyte ratio
NPV	negative predictive value
NRS	Numerical Rating Scale
O:E	observed-to-expected ratio
OOF	out-of-fold
PINP	procollagen type I N-terminal propeptide
PLR	platelet-to-lymphocyte ratio
PPV	positive predictive value
PRI	Pelvic Ring Instability score
PTH	parathyroid hormone
RF	random forest
SI	sacroiliac
SMD	standardised mean difference
STROBE	Strengthening the Reporting of Observational Studies in Epidemiology
TF90	90-day treatment failure
