# Supplementary material for: Explainable Multimodal Machine Learning Predicts 90-Day Treatment Failure in Older Patients with Fragility Fractures of the Pelvis
Source: J Clin Med. 2026 Aug 21;15(16):6487. doi: 10.3390/jcm15166487 (PMC13514191; doi:10.3390/jcm15166487)
Supplement: Supplementary file 1 [file jcm-15-06487-s001.zip › jcm-4434028-supplementary.pdf]

Supplementary Materials for

Explainable multimodal machine learning predicts 90-day treatment failure in older patients with fragility fractures of the pelvis

Kangwei Wang ***et al.***

*Corresponding author. Email: wengyuxiong@hust.edu.cn

This PDF file includes:

Supplementary Text
Figures S1 to S9
Tables S1 to S12

Other Supplementary Materials for this manuscript include the following:

None

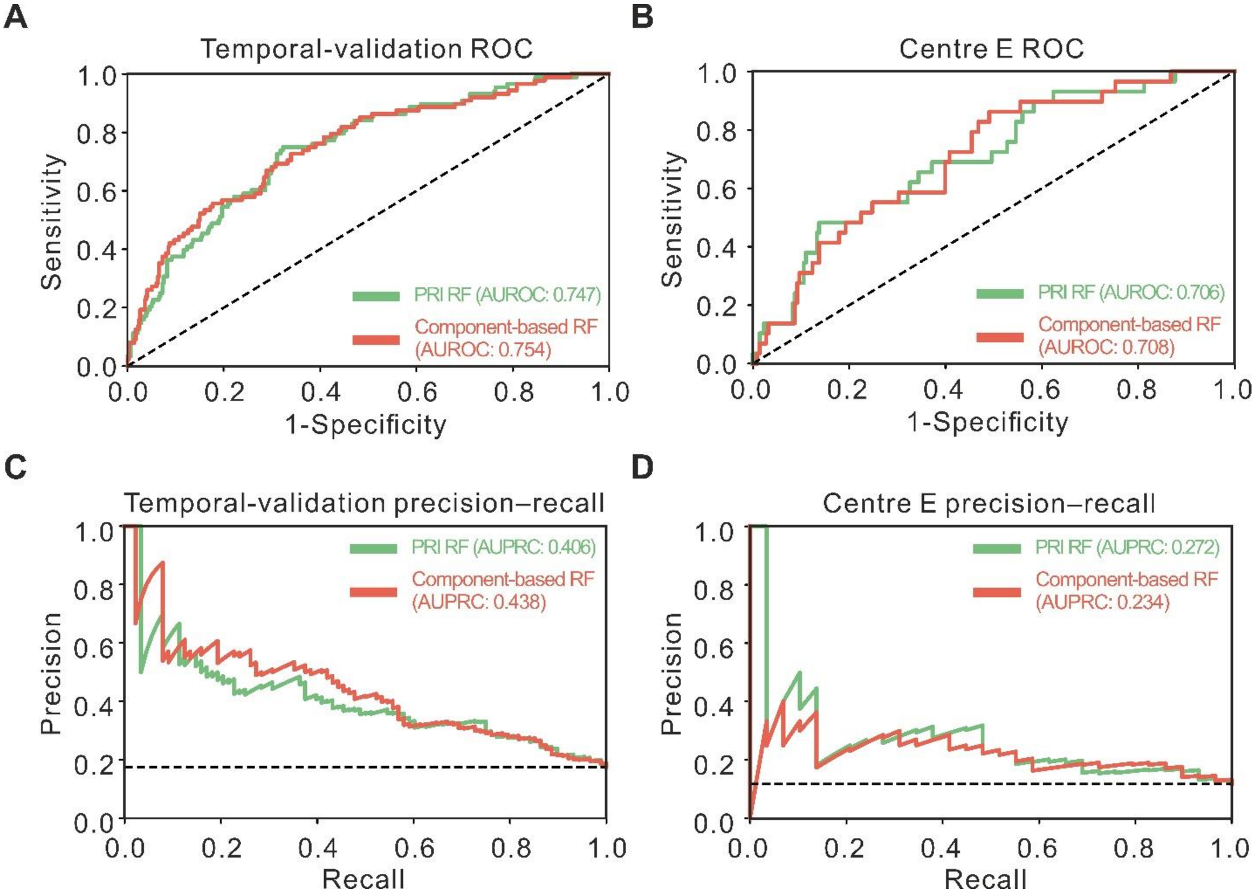

Supplementary Figure S1. Sensitivity analysis comparing alternative representations of pelvic-ring instability. **(A, B)** Receiver operating characteristic (ROC) curves comparing the PRI-score random forest (RF) with the component-based RF in the temporal-validation cohort **(A)** and Centre E internal–external validation cohort **(B)**. **(C, D)** Corresponding precision–recall curves in the temporal-validation cohort **(C)** and Centre E cohort **(D)**. AUROC, area under the receiver operating characteristic curve; AUPRC, area under the precision–recall curve; FFP, fragility fractures of the pelvis; PRI, Pelvic Ring Instability score; RF, random forest.

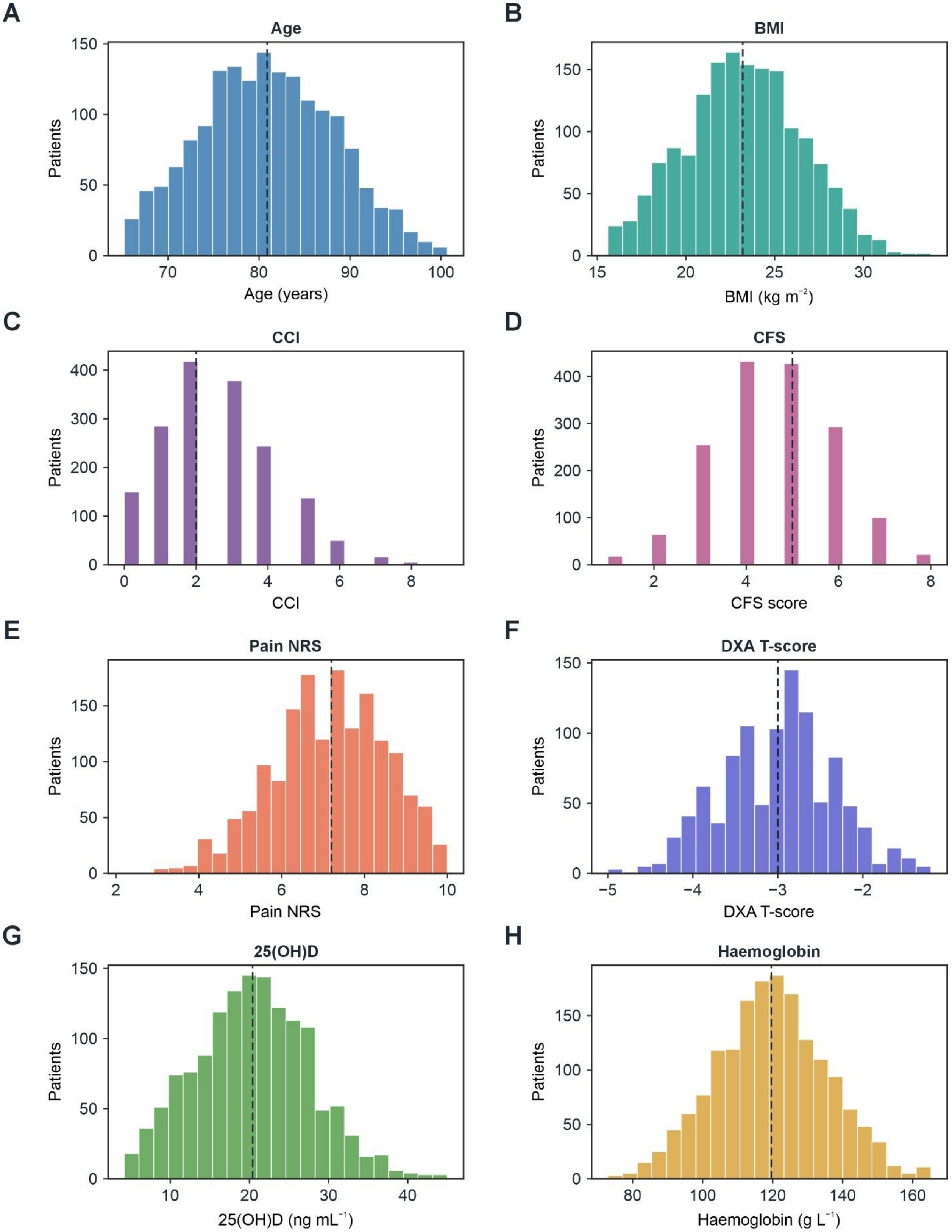

Supplementary Figure S2. Exploratory subgroup discrimination of the core random forest model in temporal validation. **(A–H)** Receiver operating characteristic curves for

the core random forest (RF) according to sex, age and FFP category in the temporal-validation cohort: women **(A)**, men **(B)**, patients aged ≥80 years **(C)**, patients aged <80 years **(D)**, FFP I **(E)**, FFP II **(F)**, FFP III **(G)** and FFP IV **(H)**. Panel annotations report the AUROC, subgroup sample size and number of TF90 events. AUROC, area under the receiver operating characteristic curve; FFP, fragility fractures of the pelvis; TF90, 90-day treatment failure.

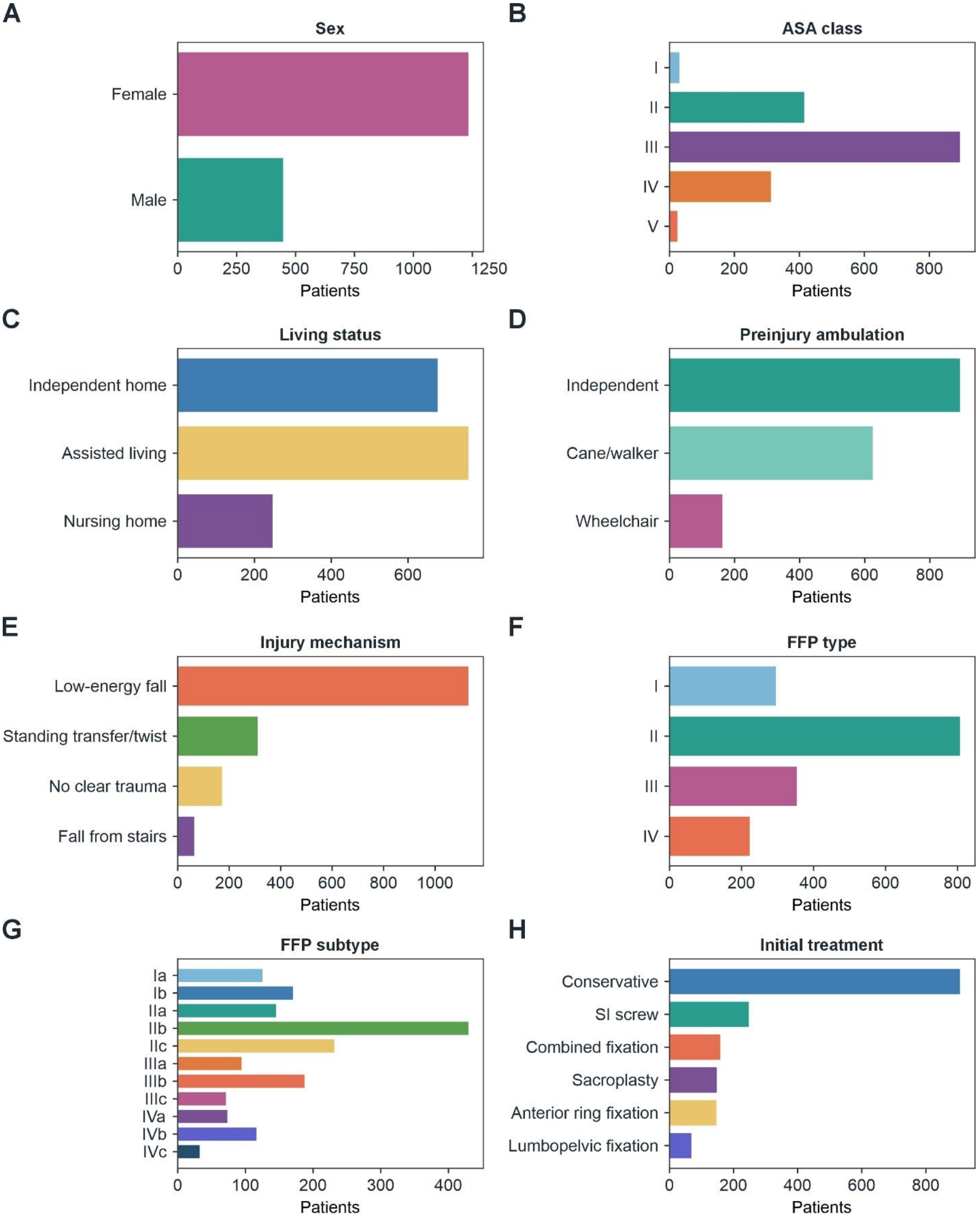

Supplementary Figure S3. Distributions of selected admission characteristics in the analytical cohort. (A–H) Frequency distributions of age **(A)**, body mass index (BMI) **(B)**, Charlson Comorbidity Index (CCI) **(C)**, Clinical Frailty Scale (CFS) score **(D)**, admission pain Numerical Rating Scale (NRS) score **(E)**, dual-energy X-ray absorptiometry (DXA)

T-score **(F)**, serum 25-hydroxyvitamin D [25(OH)D] concentration **(G)** and haemoglobin concentration **(H)**. Bar heights indicate the number of patients within each interval or score category.

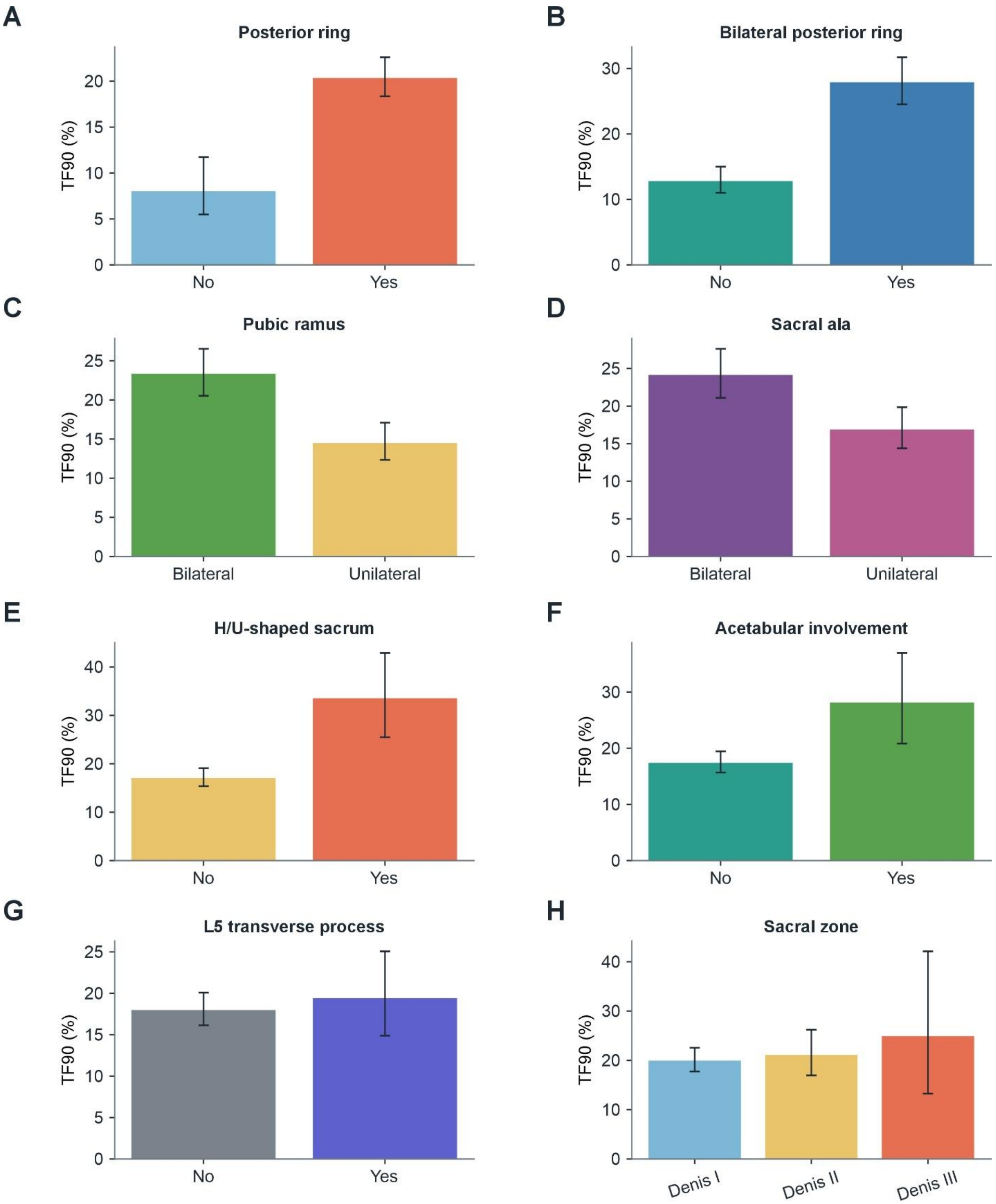

Supplementary Figure S4. Distribution of categorical clinical, functional, fracture and treatment characteristics. (A–H) Numbers of patients according to sex **(A)**, American Society of Anesthesiologists (ASA) physical-status class **(B)**, prefracture living status **(C)**, preinjury ambulatory status **(D)**, injury mechanism **(E)**, FFP category **(F)**, FFP subtype **(G)** and initial treatment strategy **(H)**. Initial treatment categories comprised conservative

management, percutaneous sacroiliac screw fixation, combined anterior–posterior fixation, sacroplasty, anterior-ring fixation and lumbopelvic fixation. Bar lengths indicate patient counts. FFP, fragility fractures of the pelvis; SI, sacroiliac.

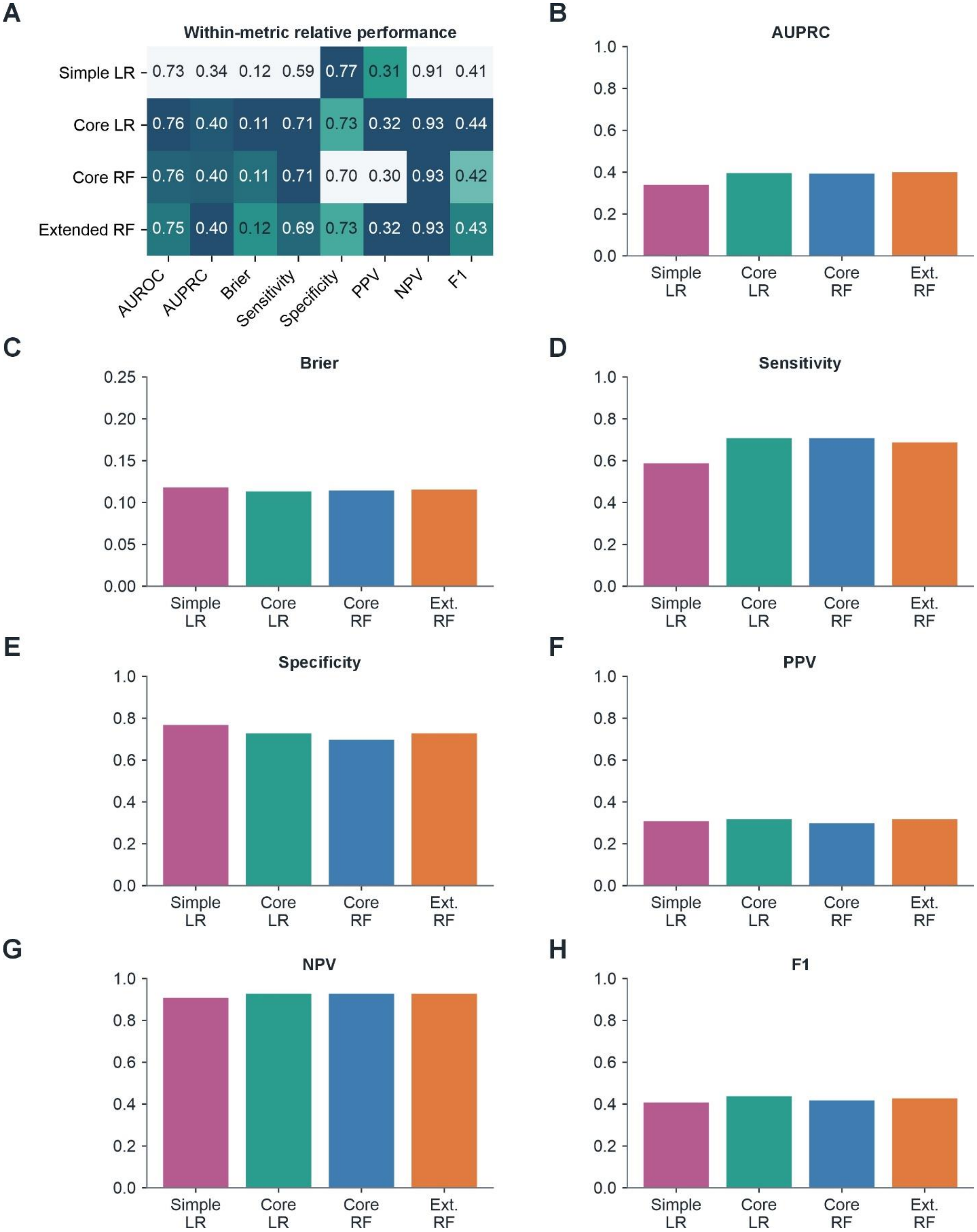

Supplementary Figure S5. Observed TF90 incidence according to fracture-morphology characteristics. (A–H) Unadjusted TF90 incidence according to posterior-ring involvement **(A)**, bilateral posterior-ring involvement **(B)**, unilateral versus bilateral

pubic-ramus fracture **(C)**, unilateral versus bilateral sacral-ala fracture **(D)**, H- or U-shaped sacral morphology **(E)**, acetabular involvement **(F)**, L5 transverse-process fracture **(G)** and Denis sacral zone **(H)**. Bars indicate observed TF90 proportions, and error bars denote 95% confidence intervals. TF90, 90-day treatment failure.

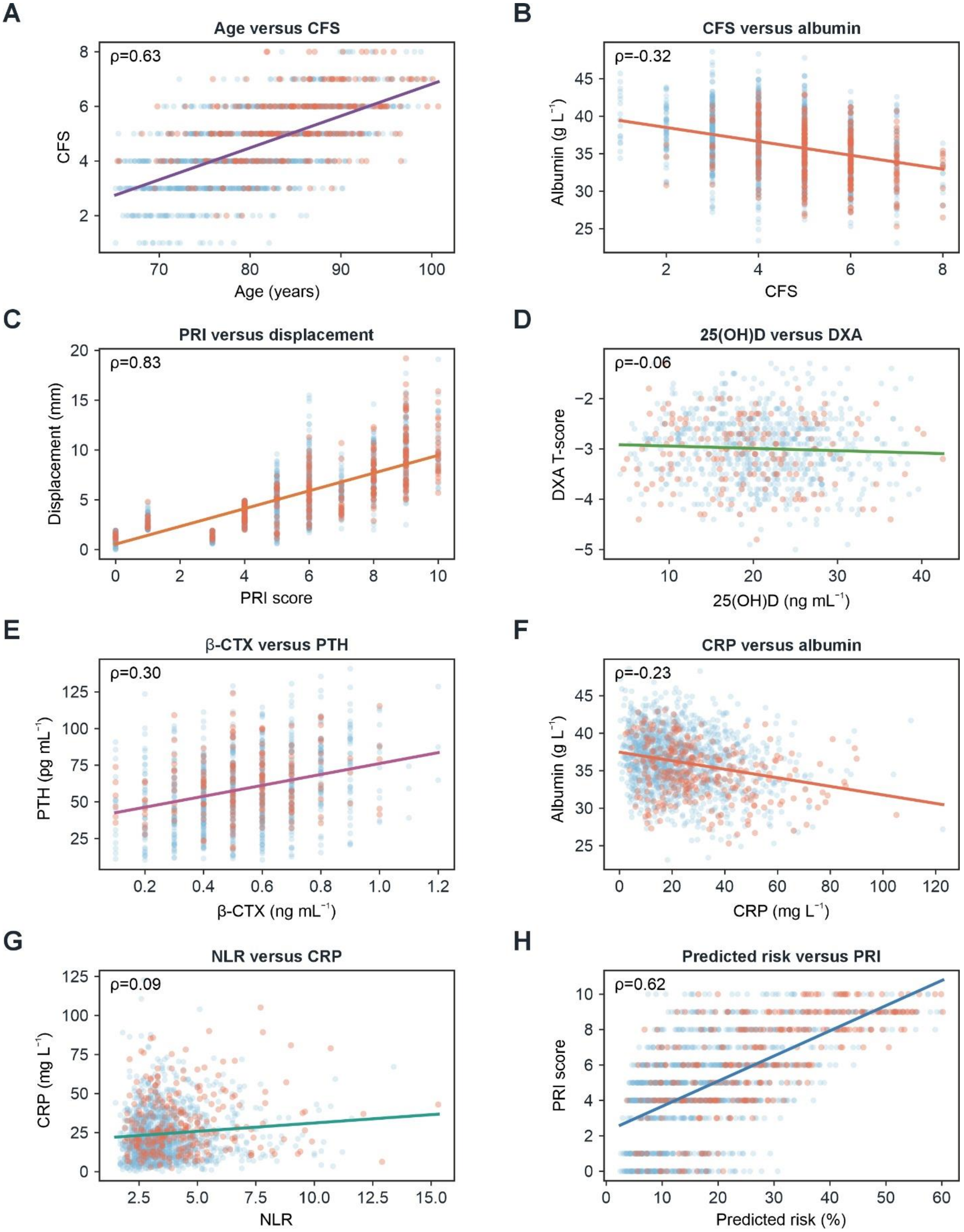

Supplementary Figure S6. Pairwise relationships among representative clinical, mechanical, inflammatory and skeletal variables. (A–H) Patient-level relationships between age and CFS **(A)**, CFS and albumin **(B)**, PRI and maximum fracture displacement

(C), serum 25(OH)D concentration and DXA T-score **(D)**, β-CTX and parathyroid hormone **(E)**, CRP and albumin **(F)**, NLR and CRP **(G)**, and core random forest predicted risk and PRI **(H)**. Points represent individual patients and are coloured according to observed TF90 status. Solid lines show fitted trends for visual description, and annotations report Spearman correlation coefficients (ϱ). CFS, Clinical Frailty Scale; CRP, C-reactive protein; DXA, dual-energy X-ray absorptiometry; NLR, neutrophil-to-lymphocyte ratio; PRI, Pelvic Ring Instability score; PTH, parathyroid hormone; TF90, 90-day treatment failure.

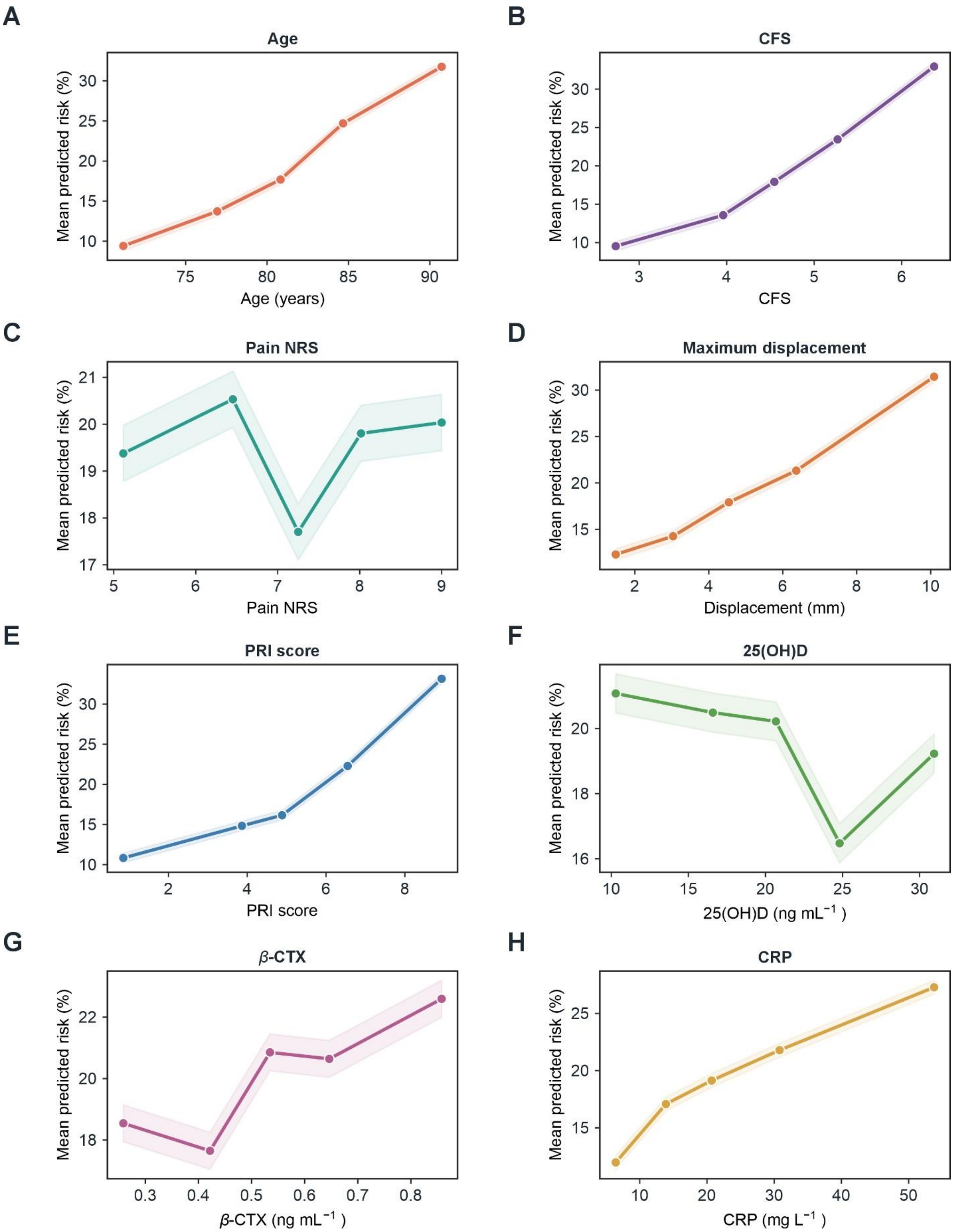

Supplementary Figure S7. Comparative performance of candidate models in temporal validation. (A) Heatmap summarising the performance of the simple logistic regression

(LR), core LR, core random forest (RF) and extended RF models across AUROC, AUPRC, Brier score, sensitivity, specificity, PPV, NPV and F1 score. Cell values report the corresponding estimates, and colour intensity represents relative performance within each metric; higher values are favourable for all metrics except the Brier score, for which lower values indicate greater probabilistic accuracy. **(B–H)** Model-specific comparisons of AUPRC **(B)**, Brier score **(C)**, sensitivity **(D)**, specificity **(E)**, PPV **(F)**, NPV **(G)** and F1 score **(H)**. AUROC, area under the receiver operating characteristic curve; AUPRC, area under the precision–recall curve; NPV, negative predictive value; PPV, positive predictive value.

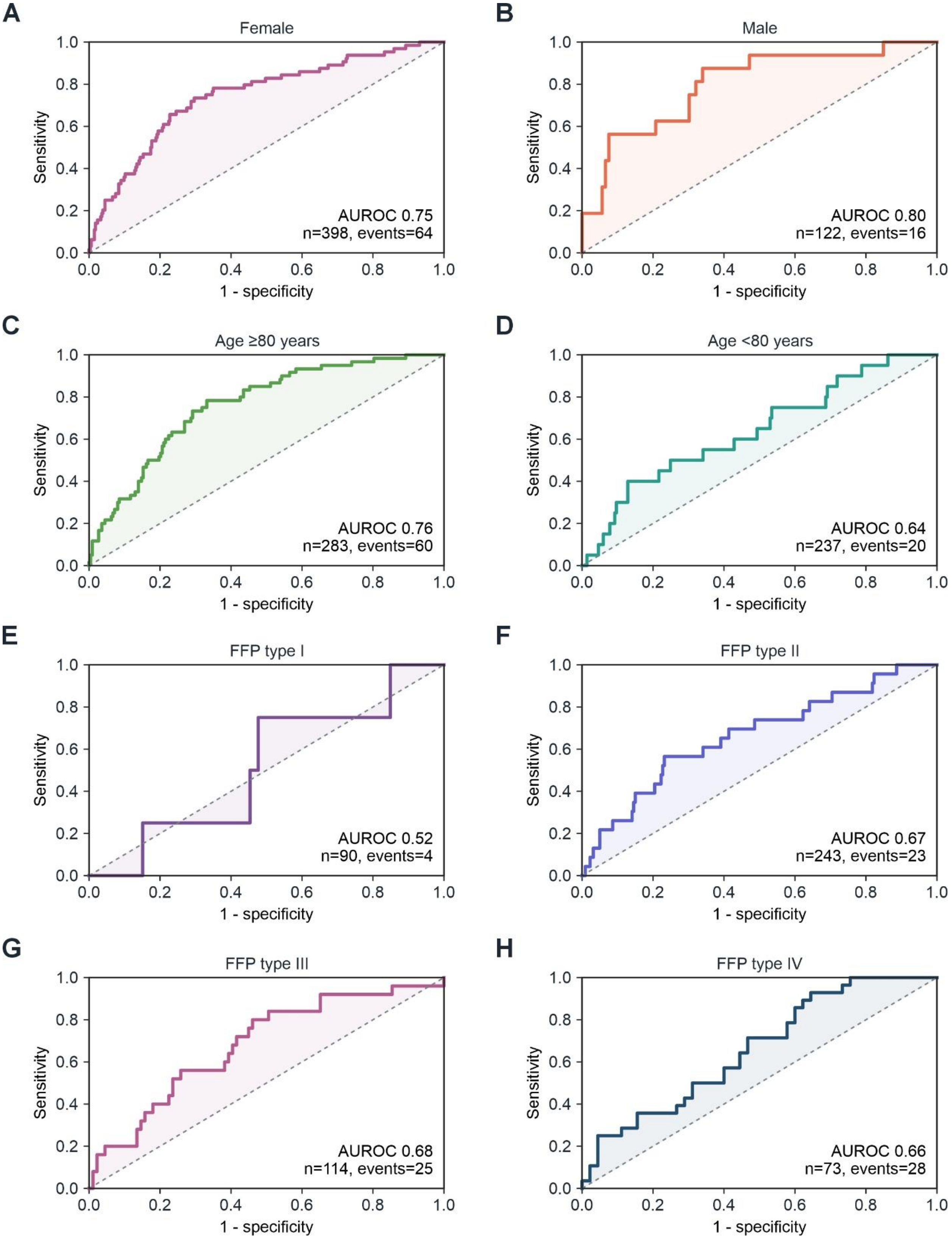

Supplementary Figure S8. Threshold-dependent operating characteristics of the core random forest in temporal validation. (A–H) Sensitivity **(A)**, specificity **(B)**, PPV **(C)**,

NPV **(D)**, F1 score **(E)**, proportion of patients classified as positive **(F)**, proportion of TF90 events captured **(G)** and model net benefit **(H)** across candidate predicted-risk thresholds in the temporal-validation cohort. NPV, negative predictive value; PPV, positive predictive value; TF90, 90-day treatment failure.

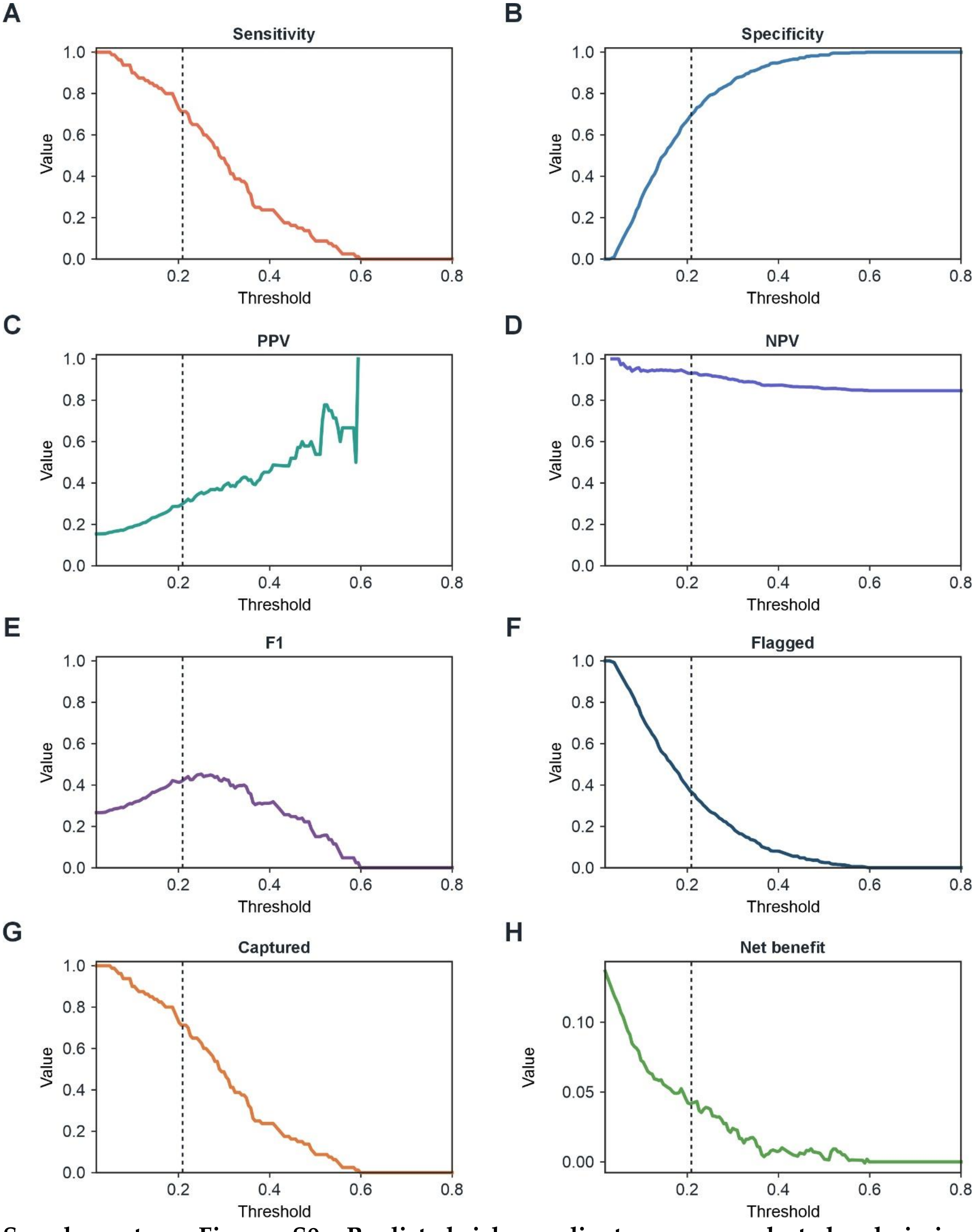

Supplementary Figure S9. Predicted-risk gradients across selected admission characteristics. (A–H) Mean core random forest predicted risk across grouped values of age **(A)**, CFS **(B)**, admission pain NRS score **(C)**, maximum fracture displacement **(D)**, PRI

(E), serum 25(OH)D concentration **(F)**, serum β-CTX concentration **(G)** and CRP concentration **(H)** in the temporal-validation cohort. Points represent mean predicted risk within each grouped interval, connecting lines depict the observed prediction gradient, and shaded bands indicate 95% confidence intervals. CFS, Clinical Frailty Scale; CRP, C-reactive protein; NRS, Numerical Rating Scale; PRI, Pelvic Ring Instability score.

Supplementary Table S1. Cohort selection and exclusions

Cohort stage	n	Comment
Records screened across five centres	5000	Starting pool
Excluded: age <65 years	612	Eligibility exclusion
Excluded: high-energy, pathological, or non-FFP fracture	534	Eligibility exclusion
Excluded: prior pelvic surgery or pelvic malignancy	241	Eligibility exclusion
Excluded: duplicate, transfer, or non-index episode	287	Episode-level exclusion
Excluded: no interpretable baseline pelvic CT completed before the ≤24 h prediction landmark	421	Required for FFP classification and PRI calculation
Excluded: treatment decision occurred before completion of baseline CT/assessment or index strategy unknown	198	Prediction chronology unavailable
Excluded: 90-day vital/treatment status not ascertainable	642	Outcome unavailable
Excluded: core eligibility or outcome-defining data unavailable	381	Cannot establish inclusion or TF90
Final analytical cohort	1684	DXA and bone biomarkers were not exclusion criteria; missing values retained and imputed within folds

Supplementary Table S2. Selected characteristics across development and validation cohorts

Characteristic	Development (n=985)	Temporal validation (n=520)	Internal–external validation (n=179)	*p*
Age, years	81.2 (7.6)	80.9 (6.9)	80.0 (6.7)	0.106
Female sex	715 (72.6)	398 (76.5)	122 (68.2)	0.065
Body mass index, kg m^{-2}	23.2 (3.3)	23.4 (3.3)	23.2 (3.3)	0.402
Clinical Frailty Scale	5.0 [4.0–6.0]	5.0 [4.0–6.0]	4.0 [4.0–5.0]	0.085
Admission pain NRS	7.1 (1.4)	7.2 (1.4)	7.2 (1.3)	0.619
Maximum fracture displacement, mm	5.0 (3.3)	5.1 (3.2)	4.7 (3.0)	0.284
Pelvic Ring Instability score	5.0 (2.8)	5.0 (2.8)	4.7 (2.7)	0.395
**FFP category, n (%)**				**0.116**
I	175 (17.8)	90 (17.3)	32 (17.9)	
II	462 (46.9)	243 (46.7)	103 (57.5)	
III	211 (21.4)	114 (21.9)	30 (16.8)	
IV	137 (13.9)	73 (14.0)	14 (7.8)	
Bilateral posterior ring involvement	363 (36.9)	174 (33.5)	60 (33.5)	0.361
C-reactive protein, mg L^{-1}	20.8 [12.5–33.0]	20.9 [12.2–34.5]	20.8 [12.4–34.4]	0.987
Albumin, g L^{-1}	35.9 (3.9)	36.3 (3.8)	36.4 (3.7)	0.062
eGFR, mL min−1 1.73 m^{-2}	68.6 (15.9)	68.5 (15.4)	69.8 (16.0)	0.635
DXA T-score	-3.0 (0.7)	-3.0 (0.7)	-3.0 (0.6)	0.566
25-hydroxyvitamin D, ng mL^{-1}	20.3 (7.2)	20.6 (7.3)	20.6 (7.2)	0.752
Initial operative treatment	487 (49.4)	220 (42.3)	69 (38.5)	0.003
Primary TF90	191 (19.4)	80 (15.4)	36 (20.1)	0.126

Supplementary Table S3. Predictor missingness and prespecified imputation procedures

Variable	Observed n	Missing n	Missing (%)	Development reference value	Extended model only
Age, years	1684	0	0.0	81.1	No
Body mass index, kg m^{-2}	1650	34	2.0	23.05	No
Charlson Comorbidity Index	1684	0	0.0	2	No
Clinical Frailty Scale	1611	73	4.3	5	No
Time from injury to admission, days	1684	0	0.0	4	No
Admission pain NRS	1652	32	1.9	7.2	No
Maximum fracture displacement, mm	1684	0	0.0	4.3	No
Pelvic Ring Instability score (0–10)	1684	0	0.0	5	No
Serum calcium, mmol L^{-1}	1663	21	1.2	2.3	No
Serum phosphate, mmol L^{-1}	1639	45	2.7	1.1	No
Alkaline phosphatase, U L^{-1}	1638	46	2.7	98.1	No
Haemoglobin, g L^{-1}	1676	8	0.5	119.2	No
White blood cell count, $\times 10^9$ L^{-1}	1672	12	0.7	7.4	No
Neutrophil count, $\times 10^9$ L^{-1}	1659	25	1.5	5.1	No
Lymphocyte count, $\times 10^9$ L^{-1}	1658	26	1.5	1.5	No
Platelet count, $\times 10^9$ L^{-1}	1674	10	0.6	228.2	No
Neutrophil-to-lymphocyte ratio	1633	51	3.0	3.4	No
Platelet-to-lymphocyte ratio	1648	36	2.1	149	No
C-reactive protein, mg L^{-1}	1621	63	3.7	20.8	No
Erythrocyte sedimentation rate, mm h^{-1}	1508	176	10.5	27.7	No
Albumin, g L^{-1}	1636	48	2.9	36	No
D-dimer, mg L^{-1}	1531	153	9.1	2.2	No
Creatinine, µmol L^{-1}	1666	18	1.1	81.05	No
eGFR, mL min−1 1.73 m^{-2}	1634	50	3.0	68.5	No
Glucose, mmol L^{-1}	1641	43	2.6	6.2	No
Sodium, mmol L^{-1}	1670	14	0.8	138.9	No
Sex	1684	0	0.0	Female	No
ASA Class	1684	0	0.0	III	No
Living Status	1684	0	0.0	Assisted living	No
Preinjury Ambulation	1684	0	0.0	Independent	No
Injury Mechanism	1684	0	0.0	Low-energy fall	No

Variable	Observed n	Missing n	Missing (%)	Development reference value	Extended model only
FFP Type	1684	0	0.0	II	No
FFP subtype	1684	0	0.0	IIb	No
Posterior Ring Involvement	1684	0	0.0	Yes	No
Bilateral Posterior Ring Involvement	1684	0	0.0	No	No
Pubic Ramus Fracture	1684	0	0.0	Unilateral	No
Sacral Ala Fracture	1684	0	0.0	Unilateral	No
Sacral fracture zone	1684	0	0.0	Denis I	No
H or U Shaped Sacral Fracture	1684	0	0.0	No	No
Acetabular Involvement	1684	0	0.0	No	No
L5 Transverse Process Fracture	1684	0	0.0	No	No
Known Osteoporosis	1684	0	0.0	Yes	No
Preinjury Antiosteoporosis Therapy	1684	0	0.0	None	No
Anticoagulant or Antiplatelet	1684	0	0.0	No	No
DXA lumbar or hip T-score	1038	646	38.4	-2.9	Yes
25-hydroxyvitamin D, ng mL^{-1}	1409	275	16.3	20.25	Yes
Parathyroid hormone, pg mL^{-1}	1319	365	21.7	58.3	Yes
Osteocalcin, ng mL^{-1}	1183	501	29.8	18.3	Yes
PINP, ng mL^{-1}	1186	498	29.6	54	Yes
β-CTX, ng mL^{-1}	1142	542	32.2	0.5	Yes

Supplementary Table S4. Operational definitions and ascertainment of the primary TF90 endpoint and its components

Endpoint	Operational definition	Ascertainment and adjudication	Assessment window
Persistent pain / immobility	Pain NRS ≥5 despite optimised analgesia or fracture-attributable inability to stand, transfer or walk 10 m with the usual aid for ≥48 h.	Clinical, rehabilitation and follow-up records; potentially qualifying events independently adjudicated by two trained reviewers blinded to model predictions.	Days 14–90; both initial treatment groups
Delayed conversion to surgery	Unplanned operative stabilisation after initial nonoperative management for persistent fracture-related pain/immobility or radiographic progression.	Operative records, clinical notes and imaging; chronology verified against the initial treatment strategy.	Days 1–90; initially nonoperative patients only
Secondary displacement / progression	New or increased pelvic-ring displacement ≥2 mm or progression to a higher FFP instability pattern relative to baseline CT.	Baseline and follow-up imaging reviewed for qualifying progression; ambiguous events adjudicated using the predefined procedure.	Days 1–90; both groups
FFP-related unplanned readmission	Unplanned admission >24 h primarily attributable to fracture-related pain, mobility failure, radiographic progression, or wound/implant complications.	Readmission records, discharge documentation and relevant clinical/imaging records.	Days 1–90; both groups
Revision surgery / reoperation	Unplanned repeat pelvic operation after index surgery or after delayed conversion.	Operative records and treatment chronology; a preceding conversion was required for initially nonoperative patients.	Days 1–90; operative or converted patients
All-cause mortality	Death from any cause within 90 days.	Hospital records and available structured follow-up/mortality documentation.	Days 1–90; both groups
Primary TF90	Occurrence of ≥1 of the six components; components were unweighted and allowed to overlap.	Derived from the adjudicated component events.	Days 1–90; pain/immobility assessed from day 14
Objective composite	Any TF90 component except persistent pain/immobility.	Derived from adjudicated component events.	Days 1–90
Major clinical-event composite	Delayed conversion, FFP-related readmission, revision/reoperation, or mortality.	Derived from adjudicated component events.	Days 1–90
Hard procedural/death endpoint	Delayed conversion, revision/reoperation, or mortality.	Derived from adjudicated component events.	Days 1–90

Supplementary Table S5. Incidence of TF90 components and sensitivity endpoints across cohorts and initial treatment strata

Endpoint	Overall (n=1684)	Development (n=985)	Temporal validation (n=520)	Centre E (n=179)	Initially nonoperative (n=908)	Initially operative (n=776)	*p* across cohorts	*p* by initial treatment
Persistent pain or immobility	191 (11.3)	118 (12.0)	49 (9.4)	24 (13.4)	110 (12.1)	81 (10.4)	0.216	0.280
Delayed conversion to surgery	83 (4.9)	52 (5.3)	23 (4.4)	8 (4.5)	83 (9.1)	0 (0.0)	0.733	NA
Secondary displacement	109 (6.5)	58 (5.9)	36 (6.9)	15 (8.4)	57 (6.3)	52 (6.7)	0.405	0.725
FFP-related readmission	95 (5.6)	66 (6.7)	21 (4.0)	8 (4.5)	42 (4.6)	53 (6.8)	0.080	0.051
Revision surgery or reoperation	93 (5.5)	57 (5.8)	27 (5.2)	9 (5.0)	20 (2.2)	73 (9.4)	0.850	<0.001
90-day mortality	8 (0.5)	7 (0.7)	1 (0.2)	0 (0.0)	7 (0.8)	1 (0.1)	0.410	0.076
**Primary TF90**	**307 (18.2)**	**191 (19.4)**	**80 (15.4)**	**36 (20.1)**	**170 (18.7)**	**137 (17.7)**	**0.126**	**0.572**
Objective composite	253 (15.0)	156 (15.8)	71 (13.7)	26 (14.5)	135 (14.9)	118 (15.2)	0.519	0.846
Major clinical event	211 (12.5)	138 (14.0)	56 (10.8)	17 (9.5)	111 (12.2)	100 (12.9)	0.084	0.683
Hard procedural/death endpoint	164 (9.7)	104 (10.6)	47 (9.0)	13 (7.3)	90 (9.9)	74 (9.5)	0.318	0.795

Supplementary Table S6. Study-specific Pelvic Ring Instability score specification

Component	Scoring rule	Minimum	Maximum	Timing or source
FFP type	I=0; II=1; III=2; IV=3	0	3	Baseline CT completed before prediction landmark
Posterior ring involvement	No=0; Yes=2	0	2	Baseline CT
Bilateral posterior ring involvement	No=0; Yes=2	0	2	Baseline CT
Maximum displacement	<2 mm=0; 2.0–4.9 mm=1; ≥5.0 mm=2	0	2	Baseline CT
H- or U-shaped sacral fracture	No=0; Yes=1	0	1	Baseline CT
**Total PRI score (PRI_Revised_0_10)**	**Sum of five components; higher score indicates greater mechanical instability**	**0**	**10**	**Derived before prediction; study-specific and not independently validated**

Supplementary Table S7. Clinical Frailty Scale assessment and observed inter-rater reliability

Assessment item	Result
Reference status	Usual pre-injury functional status approximately 2 weeks before fracture; acute admission appearance was not used
Primary assessment	Standardised CFS assessment integrating patient interview, caregiver collateral information and pre-admission clinical records
Assessor training	Structured guidance, worked examples and calibration vignettes completed before data abstraction
Outcome blinding	CFS assessors were blinded to TF90 status and model predictions
Double-rating sampling	Centre- and year-stratified sample of records with an observed primary CFS
Double-rated records, n	344
Quadratic-weighted Cohen's κ	0.863
ICC(2,1), absolute agreement	0.864
Exact agreement	63.1%
Agreement within 1 CFS point	92.7%

Supplementary Table S8. Hyperparameter tuning strategy and selected model specifications

Model	Search space	Primary selection metric	Tie-breaker	Grouping structure	Random seed	Selected parameters	Software
Simple logistic	C ∈ {0.01, 0.05, 0.10, 0.50}; L2 penalty; balanced class weighting	Mean AUPRC in fivefold grouped cross-validation	Mean AUROC	Centre × treatment year	20260628	C = 0.05	Python 3.13; scikit-learn 1.8.0
Core logistic	C ∈ {0.005, 0.02, 0.05, 0.10}; L2 penalty; balanced class weighting	Mean AUPRC in fivefold grouped cross-validation	Mean AUROC	Centre × treatment year	20260628	C = 0.005	Python 3.13; scikit-learn 1.8.0
Core RF	Number of trees ∈ {150, 300}; maximum depth ∈ {4, 6}; minimum leaf size = 8; maximum features = √p; balanced subsampling	Mean AUPRC in fivefold grouped cross-validation	Mean AUROC	Centre × treatment year	20260628	number of trees = 300; maximum depth = 4; minimum leaf size = 8; maximum features = √p	Python 3.13; scikit-learn 1.8.0
Extended RF	Core RF grid applied to core predictors plus DXA and bone-metabolism biomarkers	Mean AUPRC in fivefold grouped cross-validation	Mean AUROC	Centre × treatment year	20260628	number of trees = 300; maximum depth = 6; minimum leaf size = 8; maximum features = √p	Python 3.13; scikit-learn 1.8.0

Supplementary Table S9. Cross-validated hyperparameter search results

Model	Rank by AUPRC	Mean CV AUPRC	s.d.	Parameters
**Core RF**	**1**	**0.389**	**0.031**	**number of trees = 300; maximum depth = 4; minimum leaf size = 8; maximum features = √p**
Core RF	2	0.388	0.027	number of trees = 150; maximum depth = 4; minimum leaf size = 8; maximum features = √p
Core RF	3	0.383	0.021	number of trees = 300; maximum depth = 6; minimum leaf size = 8; maximum features = √p
Core RF	4	0.382	0.018	number of trees = 150; maximum depth = 6; minimum leaf size = 8; maximum features = √p
**Core logistic**	**1**	**0.405**	**0.038**	**C = 0.005**
Core logistic	2	0.399	0.039	C = 0.02
Core logistic	3	0.396	0.037	C = 0.05
Core logistic	4	0.391	0.036	C = 0.1
**Extended RF**	**1**	**0.389**	**0.022**	**number of trees = 300; maximum depth = 6; minimum leaf size = 8; maximum features = √p**
Extended RF	2	0.385	0.016	number of trees = 300; maximum depth = 4; minimum leaf size = 8; maximum features = √p
Extended RF	3	0.380	0.030	number of trees = 150; maximum depth = 6; minimum leaf size = 8; maximum features = √p
Extended RF	4	0.380	0.017	number of trees = 150; maximum depth = 4; minimum leaf size = 8; maximum features = √p
**Simple logistic**	**1**	**0.401**	**0.028**	**C = 0.05**
Simple logistic	2	0.401	0.015	C = 0.01
Simple logistic	3	0.399	0.028	C = 0.1
Simple logistic	4	0.396	0.030	C = 0.5

Supplementary Table S10. Permutation feature importance of the Core RF model

Rank	Predictor	Mean decrease in AUROC	s.d.	Clinical domain
1	Bilateral posterior-ring involvement	0.0423	0.0049	Pelvic-ring morphology and mechanical instability
2	Pelvic Ring Instability score	0.0395	0.0071	Integrated pelvic-ring instability
3	Neutrophil-to-lymphocyte ratio	0.0368	0.0050	Inflammatory state
4	Albumin	0.0353	0.0042	Nutritional and physiological reserve
5	FFP category	0.0339	0.0040	Pelvic-ring morphology and mechanical instability
6	Platelet-to-lymphocyte ratio	0.0317	0.0042	Inflammatory state
7	Maximum fracture displacement	0.0311	0.0068	Pelvic-ring morphology and mechanical instability
8	Erythrocyte sedimentation rate	0.0309	0.0021	Inflammatory state
9	C-reactive protein	0.0303	0.0032	Inflammatory state
10	Age	0.0298	0.0054	Demographic and physiological context
11	FFP subtype	0.0293	0.0018	Pelvic-ring morphology
12	D-dimer, mg L^{-1}	0.0289	0.0012	Haemostatic and systemic state
13	Sodium, mmol L^{-1}	0.0288	0.0001	Metabolic state
14	Lymphocyte count, $\times 10^9$ L^{-1}	0.0286	0.0017	Inflammatory and immune state
15	Sacral fracture zone	0.0285	0.0009	Pelvic-ring morphology

Supplementary Table S11. Calibration and discrimination of the Core RF model by centre, treatment year and initial treatment

Stratification	Group	N	Events, n (%)	Mean predicted risk (%)	O:E ratio	AUROC	AUPRC	Brier score	Calibration intercept	Calibration slope
**By centre**	**Centre A**	**497**	**88 (17.7)**	**20.1**	**0.88**	**0.751**	**0.413**	**0.127**	**0.14**	**1.27**
By centre	Centre B	405	71 (17.5)	18.8	0.93	0.731	0.352	0.131	0.01	1.08
By centre	Centre C	335	62 (18.5)	19.1	0.97	0.692	0.322	0.142	-0.27	0.82
By centre	Centre D	268	50 (18.7)	19.4	0.96	0.733	0.442	0.132	0.07	1.11
By centre	Centre E	179	36 (20.1)	17.7	1.14	0.745	0.375	0.145	0.37	1.14
**By treatment year**	**2015**	**98**	**15 (15.3)**	**17.8**	**0.86**	**0.674**	**0.307**	**0.126**	**-0.48**	**0.79**
By treatment year	2016	110	13 (11.8)	16.6	0.71	0.761	0.284	0.099	-0.29	1.11
By treatment year	2017	136	22 (16.2)	20.7	0.78	0.776	0.394	0.118	0.04	1.35
By treatment year	2018	132	27 (20.5)	17.9	1.14	0.756	0.484	0.141	0.66	1.35
By treatment year	2019	124	27 (21.8)	19.4	1.13	0.676	0.350	0.162	-0.12	0.78
By treatment year	2020	151	37 (24.5)	21.4	1.15	0.644	0.380	0.179	-0.29	0.60
By treatment year	2021	158	41 (25.9)	20.4	1.27	0.766	0.553	0.166	0.71	1.30
By treatment year	2022	187	32 (17.1)	19.2	0.89	0.693	0.326	0.134	-0.29	0.89
By treatment year	2023	173	24 (13.9)	18.2	0.76	0.778	0.388	0.106	0.09	1.37
By treatment year	2024	191	35 (18.3)	19.3	0.95	0.725	0.402	0.133	-0.01	1.05
By treatment year	2025	224	34 (15.2)	19.5	0.78	0.783	0.433	0.110	0.17	1.44
**By initial treatment**	**Nonoperative**	**908**	**170 (18.7)**	**16.6**	**1.13**	**0.780**	**0.470**	**0.130**	**0.87**	**1.50**
By initial treatment	Operative	776	137 (17.7)	22.3	0.79	0.691	0.335	0.138	-0.47	0.86

Supplementary Table S12. Exploratory component-specific prediction performance

TF90 component	Cohort	N	Events, n (%)	AUROC (95% CI)	AUPRC (95% CI)	Brier score (95% CI)	Calibration slope (95% CI)
Persistent pain / immobility	Development OOF	985	118 (12.0)	0.699 (0.651–0.746)	0.234 (0.185–0.299)	0.100 (0.085–0.114)	1.00 (0.73–1.29)
Persistent pain / immobility	Temporal validation	520	49 (9.4)	0.730 (0.655–0.798)	0.225 (0.154–0.331)	0.081 (0.064–0.099)	1.25 (0.80–1.68)
Persistent pain / immobility	Internal–external	179	24 (13.4)	0.730 (0.654–0.822)	0.257 (0.171–0.432)	0.112 (0.082–0.151)	1.16 (0.67–1.99)
Delayed conversion to surgery	Development OOF	985	52 (5.3)	0.661 (0.593–0.720)	0.085 (0.061–0.123)	0.049 (0.038–0.063)	1.00 (0.56–1.45)
Delayed conversion to surgery	Temporal validation	520	23 (4.4)	0.713 (0.612–0.806)	0.127 (0.065–0.246)	0.041 (0.027–0.057)	1.64 (0.82–2.49)
Delayed conversion to surgery	Internal–external	179	8 (4.5)	0.465 (0.298–0.656)	0.064 (0.021–0.225)	0.043 (0.018–0.068)	-0.08 (-1.38–1.28)
Secondary displacement	Development OOF	985	58 (5.9)	0.732 (0.679–0.797)	0.114 (0.089–0.171)	0.054 (0.042–0.067)	1.00 (0.76–1.35)
Secondary displacement	Temporal validation	520	36 (6.9)	0.729 (0.639–0.806)	0.169 (0.114–0.286)	0.062 (0.043–0.078)	1.07 (0.66–1.49)
Secondary displacement	Internal–external	179	15 (8.4)	0.625 (0.503–0.723)	0.111 (0.059–0.180)	0.079 (0.042–0.120)	0.42 (-0.06–0.87)
FFP-related readmission	Development OOF	985	66 (6.7)	0.669 (0.617–0.719)	0.120 (0.083–0.188)	0.061 (0.048–0.075)	1.00 (0.68–1.36)
FFP-related readmission	Temporal validation	520	21 (4.0)	0.683 (0.583–0.784)	0.074 (0.040–0.130)	0.039 (0.027–0.053)	0.98 (0.43–1.70)
FFP-related readmission	Internal–external	179	8 (4.5)	0.713 (0.448–0.920)	0.145 (0.048–0.404)	0.042 (0.022–0.065)	1.48 (-0.25–3.74)
Revision surgery / reoperation	Development OOF	985	57 (5.8)	0.696 (0.626–0.781)	0.120 (0.086–0.190)	0.053 (0.043–0.066)	1.00 (0.62–1.52)
Revision surgery / reoperation	Temporal validation	520	27 (5.2)	0.832 (0.748–0.902)	0.221 (0.137–0.392)	0.046 (0.033–0.061)	1.96 (1.43–2.72)

TF90 component	Cohort	N	Events, n (%)	AUROC (95% CI)	AUPRC (95% CI)	Brier score (95% CI)	Calibration slope (95% CI)
Revision surgery / reoperation	Internal–external	179	9 (5.0)	0.707 (0.522–0.911)	0.178 (0.074–0.457)	0.046 (0.021–0.075)	1.35 (0.29–3.11)
